# Degradation Behavior, Biocompatibility, Electrochemical Performance, and Circularity Potential of Transient Batteries

**DOI:** 10.1002/advs.202004814

**Published:** 2021-05-06

**Authors:** Neeru Mittal, Alazne Ojanguren, Markus Niederberger, Erlantz Lizundia

**Affiliations:** ^1^ Laboratory for Multifunctional Materials Department of Materials ETH Zürich Vladimir‐Prelog‐Weg 5 Zürich 8093 Switzerland; ^2^ Life Cycle Thinking Group Department of Graphic Design and Engineering Projects Faculty of Engineering in Bilbao University of the Basque Country (UPV/EHU) Bilbao 48013 Spain; ^3^ BCMaterials Basque Center for Materials Applications and Nanostructures UPV/EHU Science Park Leioa 48940 Spain

**Keywords:** batteries, biodegradation, circular economy, recycling, transience

## Abstract

Transient technology seeks the development of materials, devices, or systems that undergo controlled degradation processes after a stable operation period, leaving behind harmless residues. To enable externally powered fully transient devices operating for longer periods compared to passive devices, transient batteries are needed. Albeit transient batteries are initially intended for biomedical applications, they represent an effective solution to circumvent the current contaminant leakage into the environment. Transient technology enables a more efficient recycling as it enhances material retrieval rates, limiting both human and environmental exposures to the hazardous pollutants present in conventional batteries. Little efforts are focused to catalog and understand the degradation characteristics of transient batteries. As the energy field is a property‐driven science, not only electrochemical performance but also their degradation behavior plays a pivotal role in defining the specific end‐use applications. The state‐of‐the‐art transient batteries are critically reviewed with special emphasis on the degradation mechanisms, transiency time, and biocompatibility of the released degradation products. The potential of transient batteries to change the current paradigm that considers batteries as harmful waste is highlighted. Overall, transient batteries are ready for takeoff and hold a promising future to be a frontrunner in the uptake of circular economy concepts.

## Graphical abstract

A critical discussion from the circular economy point of view about the degradation mechanisms, transiency time, and biocompatibility of transient batteries is provided.

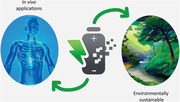

## 1 Introduction

Transient technology is a growing research area where materials, devices, or systems are able to undergo controlled degradation processes which ultimately result in their dissolution into the environment, leaving behind minimal or even nontraceable products after a period of stable operation.^[^ ^1^ ^]^ Similar to conventional nontransient analogs, transient systems must offer a stable and reliable operation during use but should then disintegrate in a programmed fashion.^[^ ^2^ ^]^ Although degradation processes may be random, to allow easy control of the transiency through external stimuli, the degradation of transient materials should preferably be activated by triggers such as pH,^[^ ^3^ ^]^ light,^[^ ^4^ ^]^ temperature,^[^ ^5^ ^]^ or the presence of specific gases/liquids.^[^ ^1^, ^6^, ^7^ ^]^ The time required for complete degradation can range from a few minutes,^[^ ^3^ ^]^ up to 45 days.^[^ ^8^ ^]^ Transient technology is rapidly gaining ground for biomedical applications, environmental sensors, or information‐sensitive hardware systems where they prevent access to data after application.^[^ ^9^ ^]^ Examples in the biomedicine include implanted medical devices (IMDs), skin‐patchable monitoring,^[^ ^10^ ^]^ wound healing systems, soft‐tissue sensing,^[^ ^11^ ^]^ or electroactive controlled release of drugs.^[^ ^12^ ^]^ In comparison with the chronic implants which are aimed to stay in the body permanently, transient medical devices can be implanted to diagnose/treat a disease and afterward disappear with no need of additional surgery for device retrieval, reducing potential risks, costs, and chronic inflammation caused by permanent devices.^[^ ^13^ ^]^ Such temporary devices are disintegrated, dissolved, resorbed, or degraded in a programmed fashion under relatively mild conditions at their end‐of‐life (EOL).^[^ ^10^, ^14^ ^]^

**Figure 1:**
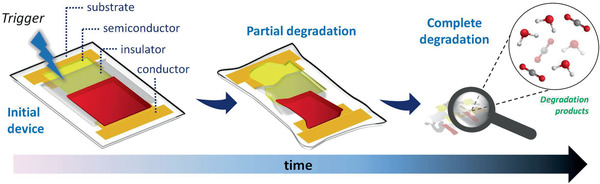
Schematic process showing the degradation of transient electronics. Once triggered, transient electronics degrade within their immediate surroundings at controlled rates to release degradation products that ideally have minimum deleterious effects on both the environment and the human body.

**Figure** **1** shows schematically the degradation of transient electronic devices.^[^ ^1^, ^2^, ^6^ ^]^ The device is usually present in the form of a 2D foil, where the active components are supported onto a mechanically flexible sheet, typically polymeric in nature. The material compositions are chosen based on both the performance and ability to degrade/dissolve with negligible associated harmful effects. Upon the application of an external trigger, a series of chemical reactions occur, and the device begins to degrade/dissolve. After a given time, all the components disappear, releasing degradation products into the medium. These products arise from the breakdown of the component materials into their respective constituents. While metals usually degrade into ionic species, polymers have the ability to degrade into soluble monomer units or oligomers (chains comprising few repeating units). As some of the biodegradation products may result toxic when exceeding certain concentrations, special care should be paid when designing transient devices.

Because of the new consumer trends, faster obsolescence, and the advent of the Internet‐of‐Things, the quantity of household and industrial electronics being consumed has continuously risen over the last years. This unprecedented increase in the use of electronic devices is leading to enormous amounts of disposed waste electrical and electronic equipment (WEEE) once electronic devices are discarded.^[^ ^15^, ^16^ ^]^ WEEE generation and management seriously jeopardize sustainability and it is considered as one of the foremost environmental issues in different countries.^[^ ^17^ ^]^ Additionally, the multicomponent character of WEEE comprising noble, ferrous/nonferrous metals, plastic, glass, and toxic chemicals, together with their mechanically/chemically durable nature seriously threatens human and environmental safety. In this scenario, many countries have implemented different policy approaches to face WEEE, where European Community Directive 2012/19/EU and RoHS Directive 2011/65/EU (Restriction of Hazardous Substances in Electrical and Electronic Equipment) are the flagship policies.^[^ ^18^ ^]^

Recycling of electronic goods can be pursued as an alternative to the increasingly pressing issue of WEEE accumulation in the environment. However, due to the applied mechanical and thermal treatments during recycling together with the accumulation of a wide variety of elements, recycling often results in downcycling, yielding goods with poorer functionalities in comparison with the original material.^[^ ^19^ ^]^ As a result, some materials can be only recycled up to 5 times before their quality decreases to the point where it can no longer be used. Moreover, despite the huge economical investment into the development and promotion of recycling, not all the regions worldwide have proper waste management policies and infrastructures. Also, collecting recyclable materials can be a real issue as they are not disposed correctly by consumers or there may be a lack of enough collection points. As a result, current recycling activities are not able to keep up with the pace of the global growth of WEEE, and only 17.4% of the generated WEEE in 2019 was properly collected and recycled.^[^ ^20^ ^]^ The whereabouts of the remaining surplus raises serious environmental concerns as a large fraction of such untraced material is probably mixed with other waste streams and directly disposed into oceans or landfills, seriously threatening the balance of our ecosystem.^[^ ^21^ ^]^ This is even more exacerbated in regions such as Africa, Oceania, or the Americas, which have WEEE recycling rates of 0.9%, 8.8%, and 9.4%, respectively.^[^ ^20^ ^]^ If new policies and technologies directed to face the large quantity of untraced WEEE are not properly applied, this dilemma could be aggravated in the near future as the global WEEE production is expected to increase from 7.3 kg per capita in 2019 to 9 kg by 2030.^[^ ^20^ ^]^

Considering that the electrochemical energy storage systems represent the most hazardous components of WEEE, new strategies are required to manage discarded batteries. The application of biodegradable materials into transient electronic devices could bypass the environmental burdens associated with the often complex, expensive, and labor‐intensive collecting, mechanical sorting, and recycling of WEEE as they can be simply degraded in the environment with no harmful effects.^[^ ^14^ ^]^ That way, transient materials are incorporated into the earth's biogeochemical cycles through biodegradation, balancing the overall carbon cycle. When intended to be recycled, transient batteries can facilitate the process as the readily degradable character of the battery encasing avoids the first manual disassembly step. The chemical recycling process to extract nonmetal fractions is also simplified as straightforward processes involving aqueous extraction can be applied to many natural polymer electrolytes (as they present easily tunable solubility properties).^[^ ^22^ ^]^ Additionally, biopolymers such as lignin have been proven to efficiently recover Co^2+^ from a leaching solution through adsorption,^[^ ^23^ ^]^ avoiding the need of hydro‐ or pyrometallurgical approaches involving harsh experimental conditions. Interestingly, these materials can be then carbonized to yield electrodes that can be reused, providing novel cues toward upcycling.^[^ ^24^ ^]^ Overall, transiency offers new possibilities for recycling as the dissolution/degradation of a given component facilitates the recovery of other materials, reaching retrieval rates as high as 96%.^[^ ^25^ ^]^ Not only recycling strategies but also material scarcity and safety play a pivotal role toward sustainable battery design. Transient batteries contain cathodes based on nontoxic and relatively earth‐abundant elements such as zinc, molybdenum, or naturally occurring bioactive compounds such as melanin. This is in sharp contrast to the widespread use of critical raw materials such as cobalt in the energy storage field, which present serious issues in the supply chain. Therefore, transient batteries represent an opportunity to shift from the use of scarce materials to greener solutions, which is an urgent task as the global demand of cobalt is expected to increase by 60 times by 2030 considering the key role of batteries to store renewable energy and the accelerated electric vehicle adoption during the COVID‐19 pandemic.^[^ ^26^ ^]^

Most of the efforts carried out till date have been devoted to transient electronics (sensors, inverters, actuators, energy harvesters, imaging devices, etc.), providing good examples of the potential of transient technology.^[^ ^27^, ^28^, ^29^ ^]^ Due to tight dimension constraints (IMDs such as pacemakers have a preferred maximum volume of 1 cm^3^),^[^ ^30^ ^]^ the implementation of transient devices for biomedical applications has been limited. In principle, wireless power transfer technology can be applied in small transient IMDs as a means to transmit electrical energy through electromagnetic fields.^[^ ^31^ ^]^ Different IMDs have been powered operating in the megahertz region, reaching input powers of a few Watts. However, power transfer efficiency is below 50% (even below 3% in many cases), transmitter–receiver distance is usually limited to 100 mm, and more importantly, the surrounding tissue of the IMD being powered may suffer undesired heating issues. Therefore, IMDs with directly connected batteries are preferred.

The miniaturization of IMDs has been delayed because of the challenge to reduce the battery size, where a bulky and durable packaging is needed to avoid the contact of the toxic battery components with the body. In order to enable fully autonomous transient devices that do not rely on external power sources, the development of transient energy storage systems plays a pivotal role. Miniaturization of transient batteries, for example, could enable self‐powered bioimplants as shown by Wallace and co‐workers, who reported a bioimplantable magnesium‐based transient battery with a thickness of just 300 µm (whole battery volume of 30 mm^3^) and a power density high enough to drive a cardiac pacemaker.^[^ ^32^ ^]^ This is in contrast to transient batteries that are not aimed for biomedical applications, where the less stringent dimension requirements allow battery volumes up to 512 mm^3^ (16 × 16 × 2 mm).^[^ ^33^ ^]^

Electrochemical energy storage systems in general, and batteries in particular, either primary (nonrechargeable) or secondary (rechargeable), are especially attractive as they outperform other energy storage systems in terms of energy density and energy conversion efficiency.^[^ ^34^, ^35^ ^]^ To date, however, transient power sources remain a major obstacle toward self‐powered transient devices and only very few works have accomplished the design, fabrication, and successful utilization of transient batteries. As one of the most representative battery types, the potential of lithium‐ion batteries (LIBs) in transient energy storage systems was investigated by Fu et al., who reported a transient LIB with a capacity of 3 mAh cm^−2^ and a working voltage above 2.0 V, which was able to degrade under alkaline conditions in aqueous potassium hydroxide solution.^[^ ^3^ ^]^

**Figure 2:**
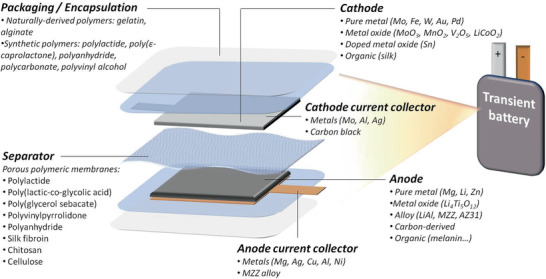
Scheme summarizing the typical architecture and most widely used materials in transient batteries (both primary and secondary). Current collectors, anode, cathode, separator, and external packaging/encapsulation are shown.

The fundamental operation of transient batteries is similar to durable batteries and relies on the conversion of chemical energy to electrical energy.^[^ ^2^, ^36^ ^]^ As schematically summarized in **Figure** **2**, transient batteries are built up by a positive electrode (cathode), a negative electrode (anode), a physical separator, two current collectors, and an external encapsulation or packaging. Although not all transient batteries present the same characteristics, generally, the anode and cathode are composed of an active material mixed with a highly conducting additive and a binder that holds all the constituents together.^[^ ^36^, ^37^, ^38^ ^]^ The separator, on the contrary, is not considered as an active part but serves to physically isolate both electrodes while enabling ionic conduction between them. The separator is usually found in the form of a porous solid membrane soaked with a liquid electrolyte, or as a gel. Current collectors enable the flow of the electrical current from the positive to the negative terminal through the device that is being powered. Finally, an external encapsulation is required to provide a protecting coating that avoids parasitic corrosion or damage of the other battery components.

Conventional secondary batteries based on lithium,^[^ ^39^ ^]^ sodium,^[^ ^40^ ^]^ aluminum,^[^ ^41^ ^]^ magnesium,^[^ ^42^ ^]^ or zinc‐ion chemistries^[^ ^43^ ^]^ are usually composed of chemically stable carbon nanomaterials/metals/metal oxides as electrodes and current collectors, nondegradable polymers as separators, organic liquids as electrolytes, and the full cell is protected by a metallic casing for high safety assurance. In this context, the potential environmental and human health effects of secondary batteries were studied using life cycle impact assessment, concluding that such batteries can be classified as hazardous due to their excessive contents of lead, cobalt, nickel, copper, chromium, thallium, and flammable electrolytes.^[^ ^44^ ^]^ Therefore, limiting both human and environmental exposures to these hazardous pollutants should be a priority, particularly in those regions of the world that lack adequate infrastructure for battery waste collection, sorting, and recycling.

Transient batteries relying on green materials may offer a promising alternative. This requires a major paradigm shift in the design of batteries. While conventional batteries are designed for being long‐lasting, degradable batteries are just the opposite. Active materials (anode, cathode), the separator/electrolyte pair, and current collectors need to be rethought so they can be degraded once a predetermined external trigger is applied. Additionally, battery packaging/casing should be redesigned as its task of permanently protecting the battery cell from external influences is no longer given. Instead of simply isolating the anode, cathode, separator, and electrolyte from the surrounding medium, the encasing should allow interaction with the surrounding environment (liquid, soil, bacteria, etc.) when necessary. Interestingly, when batteries are intended for recycling, the degradation of the battery components can even help to recover the materials more efficiently.^[^ ^25^ ^]^ Another advantage of transient batteries is that they can be degraded “on‐site” with no harmful effects, avoiding the need for often overly tedious, time‐consuming, and expensive waste collection and processing.^[^ ^45^ ^]^ As the choice of materials fulfilling these stringent requirements is mostly limited to degradable and nontoxic chemistries, most of the transient batteries reported so far suffer from low open‐circuit voltage (*V*_OC_), low power densities, and short lifetimes, failing to provide enough power for practical applications.

It is clear that material choices and design considerations for transient batteries are sharply distinct from those required for conventional batteries (either aimed or not for biomedical applications). Depending on the intended application, full battery degradation may or may not be necessary. In the case of batteries powering biomedical devices such as cardiac pacemakers or electrocardiogram signal detectors, all the components should be completely degraded in few days into monomers (for polymeric parts) or ions (active materials, mostly metals and metal oxides) that could be absorbed or excreted by the body with no toxic response. The complete degradation is an essential requirement as the accumulation of certain battery components may result in undesired biological responses (tissue inflammation, unwanted immune reactions, or wound healing delay).^[^ ^46^ ^]^ By contrast, batteries intended for environmental applications are mainly aimed at reducing the contaminant accumulation in the environment from conventional batteries, so a partial disintegration is acceptable. In this context, a quick transiency (from seconds to few minutes) is preferred for batteries powering secured hardware to prevent unauthorized access, while batteries for consumer electronics survive for a longer period of time in the environment. However, to avoid waste accumulation into marine and land environments, these transiency times should ideally be below 1 year. To further compare such requirements, which in turn define the material selection and design, the different degradation and electrochemical characteristics of conventional batteries and transient batteries are listed in **Table** **1**.

**Table 1:** Requirements for material selection and battery design regarding conventional and transient batteries. N.R.: not reported

	Requirements for material selection and battery design	Conventional batteries	Conventional medical batteries^[^ ^48^ ^]^	Transient batteries	
				Biomedical applications	Environmental applications
Degradation characteristics	Degree of degradation and degradation time	Nondegradable	Nondegradable	Full degradation into monomers/ions; approximately minutes to days	Partial degradation acceptable; seconds to years depending on the application
	Degradation products	Toxic, comprising critical raw materials	Toxic	Nontoxic, biocompatible	Barely toxic, recyclable
	Biological impact	Large	N.R.	Minor, slight inflammatory issues	Small, possible eutrophication
Electrochemical performance	Working voltage [V]	2.0–4.5	2.8–3.9	0.31–1.33	0.5–2.8
	Specific capacity [mAh g^−1^]	≤ 250	N.R. (0.8–2 Ah)	10–1060	≤150
	Energy density [Wh kg^−1^]	>150 (LIBs)^[^ ^47^ ^]^	149–200^[^ ^48^, ^49^ ^]^	16.6–694^[^ ^49^ ^]^	≤480 (Li/V_2_O_5_)
	Lifetime	Approximately years	Approximately years	3–1800 h	Up to 200 cycles

Because of their degradability, transient batteries can provide new approaches toward a “circular economy,” which establishes a framework for an economy that is restorative and regenerative by design (Ellen MacArthur Foundation). Although the circular economy is still an area open to debate, it is generally accepted that “the circular economy seeks to break away from the linear economy characterized by “make, use, dispose” in favor of a more circular model based on “reuse, recycle, or biodegrade” (Bio‐based Industries Consortium).” Similarly, in the words of the European Compost Network, “Recycling biodegradable wastes and resource efficiency lie at the heart of the circular economy.”^[^ ^50^ ^]^ Even if biodegradable/compostable materials cannot be considered as the ultimate remedy,^[^ ^51^ ^]^ such materials can support the transition to a circular economy as they create harmless secondary products (soluble oligomers and metal ions in the case of transient batteries), thus merging efficient resource utilization, value creation, and economic growth.

**Figure 3:**
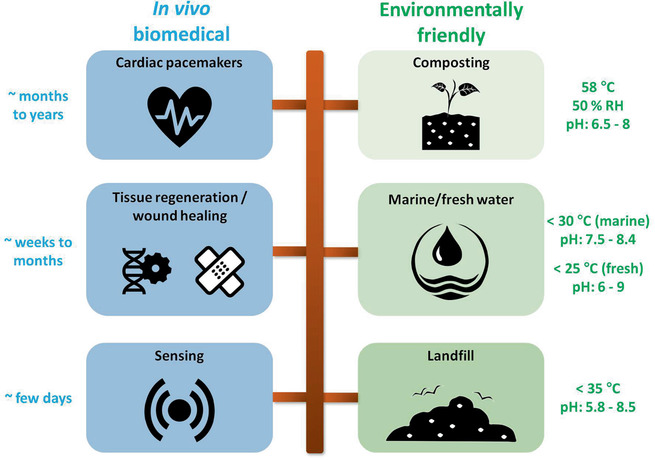
Operating and transiency time frames depending on the intended application, categorized into in vivo biomedical and environmentally friendly fields. Characteristics of degradation media may vary notably when discarded into the environment.

Apart from the electrochemical performance, one of the current bottlenecks for efficient transient batteries is their relatively slow transiency rates originating from the sluggish chemical reactions occurring between the battery constituents and the environment (generally a solvent, a cell culture media, or soil). However, as summarized in **Figure** **3**, it should be noted that different operating time frames and degradation rates are desired depending on the intended application. Long in vivo transience times of several months‐to‐years are perfectly suited for biomedical devices with medium‐term use, such as cardiac pacemakers,^[^ ^52^ ^]^ but may not be desirable at all for devices intended to perform stimulation functions for short‐term usage, such as tissue regeneration or wound healing (a few weeks).^[^ ^12^ ^]^ In fact, to effectively remove the device once it has served its purpose, fast in vivo transiency rates are needed for other biomedical applications such as electrocardiogram signal detectors, which usually operate for just a few days.^[^ ^12^ ^]^ Apart from the biomedical applications, transient batteries can be also applied to reduce the environmental burdens caused by conventional electrochemical energy storage systems. In those ex vivo applications, the transiency characteristics are mainly driven by the time that the device should function. In this context, degradation under controlled composting aerobic conditions at 58 °C, 50% of humidity, and pH of 6.5–8 (International Organization for Standardization (ISO) 20200:2015 for plastic materials) is typically fast. By contrast, many compostable polymers are not degradable (or their kinetics are extremely slow) in marine (<30 °C, pH: 7.5–8.4), fresh water (<25 °C, pH: 6–9), or even landfill (<35 °C, pH: 5.8–8.5) environments.^[^ ^53^ ^]^

Moreover, addressing the potential impact of the transient batteries during their degradation under regulated conditions (i.e., in vitro test for hydrolysis or enzymatic processes using well‐controlled conditions) and unregulated conditions such as in rivers or marine environments is of pivotal relevance toward the full understanding of how transient batteries could provide an environmentally respectful solution to our society. In this sense, there is an urgent need for a comprehensive understanding of the degradation mechanisms involving transient batteries as well as cataloging their degradation characteristics. Recognizing how transient batteries degrade and which are the resulting degradation products may help researchers to design and apply appropriate materials for transiency.

Although several works have reviewed the recent progress made on transient electronics,^[^ ^6^, ^7^, ^54^ ^]^ few efforts have been devoted to addressing the transient energy storage devices. In particular, no works have summarized the degradation characteristics of transient batteries, which is of prime interest to exploit the full potential of transient devices. Accordingly, this work critically reviews the progress made on transient batteries, covering key aspects of their degradation in terms of the governing mechanisms, degradation kinetics, and composition of the products. The effect of the degradation products of the anode, cathode, separator, electrolyte, current collectors, and encapsulation on both the human body and environment are comprehensively addressed. Their assembly and electrochemical performance in terms of energy density, specific capacity, working voltage, Coulombic efficiency (CE), and lifetime are analyzed. Along with the biomedical applications, the potential of transient technology for implementation into a circular economy perspective is highlighted. Finally, we provide an outlook on the current challenges and future requirements for transient batteries.

## 2 Degradation Behavior: Mechanism and Kinetics

Depending on the application field, different definitions of biodegradability have been provided in the literature. From a transient electronics perspective, biodegradable can be assigned to a material that is able to decompose in a physiological environment (either by microorganisms, cells, proteins, water, or ions) into constituents of lower molecular weight after a specified period of time.^[^ ^6^, ^55^ ^]^ It should be noted that not all the battery components need to be biodegradable, as fading by mere dissolution or disintegration is also enough to provide the transiency.^[^ ^56^ ^]^ During biodegradation processes, the starting material is transformed into H_2_O, CO_2_, CH_4_, and biomass, thus closing the loop back to nature.^[^ ^53^ ^]^

**Figure 4:**
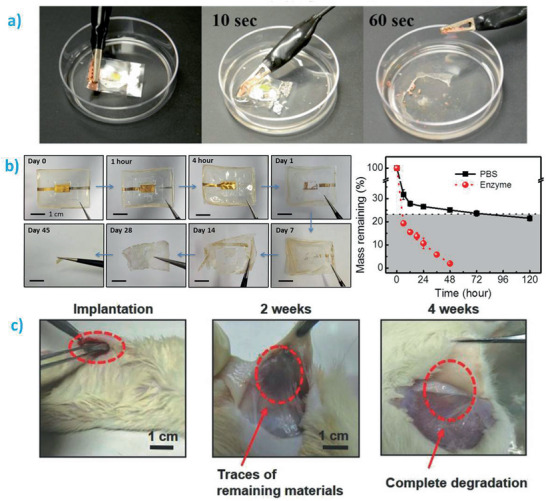
Transiency in batteries. a) Optical photographs showing the quick degradation of a LIB in water at room temperature. Reproduced with permission.^[^ ^2^ ^]^ Copyright 2015, American Chemical Society. b) Left: optical images showing the degradation of an encapsulated Mg‐based battery (36 × 27 × 0.17 mm^3^) in phosphate‐buffered saline at 37 °C. Right: mass loss of the silk fibroin–[Ch][NO_3_] gel polymer electrolyte in protease XIV solution and phosphate‐buffered saline. The dashed line accounts for the content of silk fibroin within the gel polymer electrolyte. Reproduced with permission.^[^ ^8^ ^]^ Copyright 2017, American Chemical Society. c) Degradation and biocompatibility of a transient battery showing in vivo degradation of the battery in the subcutaneous area of rats at different time periods. Complete degradation occurs in 4 weeks. Reproduced with permission.^[^ ^12^ ^]^ Copyright 2018, Wiley‐VCH.

In the following sections, the transient behavior of batteries, both primary and secondary, is analyzed. The first section is dedicated to the nonactive parts of the batteries, the packaging and the separator, while the second section is devoted to active materials (anode, cathode) and current collectors. So far, transiency times for batteries ranging from 1 min (**Figure** **4**a)^[^ ^2^ ^]^ to 45 days (Figure 4b‐left)^[^ ^8^ ^]^ have been reported. Most of the works qualitatively estimate the transiency behavior through naked eyes only, although some of them went a step further to quantitatively obtain the degradation profile of the battery (or at least, of the separator) as shown in Figure 4b‐right.^[^ ^8^ ^]^ To investigate the possible toxicity of the degradation products, other works conducted in vivo degradation studies (Figure 4c).^[^ ^12^ ^]^ Based on all those results, here we catalog the transience behavior of batteries in **Tables** **2** and **3** for nonactive and active components, respectively. The degradation rate/time of each material in a given medium (liquid solution type, pH, and temperature), degradation mechanisms, the formed products, and their main biological impacts are summarized.

**Table 2:** Transiency of polymers applied as battery separators or packaging. The degradation rate of polymers is usually reported in terms of weight loss for a given period of time. N.R.: not reported

Material	Format	Degrading medium	Rate/time	Mechanism	Products	Biological impact
PLA^[^ ^33^, ^71^, ^72^ ^]^	Packaging (*⌀* 25 and 2 mm thick disk)	Water (pH = 5.4) at 70 °C	50 wt% loss in 8 days	Hydrolysis of ester bonds, bulk erosion	Lactic acid, CO_2_, H_2_O	Nontoxic, metabolized by the body and ejected through urine and breath
		Alkaline media (pH = 9) at 70 °C	70 wt% loss in 10 days			
		Acidic media (pH = 1) at 70 °C	50 wt% loss in 10 days			
PLGA^[^ ^12^, ^76^ ^]^	Separator (300 µm thick film)	PBS (pH 7.4, 37 °C)	For a 50:50 LA/GA ratio: 20 wt% loss in 10 days	Hydrolysis of ester linkages, bulk erosion	Lactic and glycolic acids, CO_2_, H_2_O	Nontoxic, biocompatible: easy assimilation/transformation by Krebs cycle
PCL^[^ ^49^, ^77^, ^78^ ^]^	Packaging (150 µm thick film)	PBS (pH 7.4, 37 °C)	3.3 wt% loss in 90 days	Hydrolytic cleavage of ester groups, bulk erosion	Caproic acid	Nontoxic, biocompatible
		SBF (pH 7.4, 37 °C)	2.3 wt% loss in 90 days			
PGS^[^ ^79^ ^]^	Separator In vitro: 5 × 5 × 2 mm film In vivo: 6 × 6 × 3 mm film	PBS (pH 7.4, 37 °C)	17.6 wt% loss in 60 days	Cleavage of the ester linkages, surface erosion	Sebacic acid and glycerol	Nontoxic, degradation products are metabolized by the human body
		In vivo (subcutaneous area of Sprague‐Dawley rats)	100 wt% loss in 60 days			
Polyanhydride^[^ ^12^, ^15^, ^74^, ^81^ ^]^	Packaging (124 µm thick)	PBS (pH 7.4, 37 °C)	9 days	Hydrolysis of anhydride bond, surface erosion	Diacid monomers	Nontoxic
PVP^[^ ^2^, ^82^, ^94^ ^]^	Separator (nonwoven nanofiber mat, 6 × 6 cm^2^)	Aqueous media (pH 5.8, 25 °C)	100 wt% loss in 10 min	Dissolution	Dissolved PVP	Nontoxic, biocompatible
PVA^[^ ^82^, ^95^ ^]^	Packaging (40 µm thick)	Alkaline media (pH 14, 25 °C)	100 wt% loss in 30 s	Dissolution	Dissolved PVA	Quickly removed from the body; limited adverse effects, not mutagenic, not genotoxic, not carcinogenic
Polycarbonate^[^ ^3^, ^96^ ^]^	Packaging (spray‐coated, thickness N.R.)	Alkaline media (pH 14, 25 °C)	100 wt% loss in 3 min	Hydrolysis at the surface	Bisphenol A, CO_2_	Adverse health effects including altered behavior/obesity, reproductive abnormalities, cancer
Silk fibroin^[^ ^8^, ^88^, ^89^ ^]^	Separator (+choline nitrate) (55–80 µm thick)	Buffered protease XIV at 37 °C	89 wt% loss in 24 h	Enzymatic degradation, surface erosion	Amino acids	Biocompatible, noninflammatory degradation products
Chitosan^[^ ^32^, ^90^ ^]^	Separator (with choline nitrate IL) (60–100 µm thick)	PBS (pH 7.4, 37 °C)	DD > 95% cannot be degraded	Enzymatic hydrolysis by lysozyme enzymes	*N*‐acetylglucosamine, glucosamine	Biocompatible, lack of immunogenicity and inflammation
Gelatin^[^ ^11^, ^91^, ^97^ ^]^	Packaging (thickness N.R.)	Deionized water (pH 5.8, 37 °C)	Depends on cross‐linking	Hydrolysis of peptide bonds and cross‐links	N‐terminal amino acids	Nontoxic
Cellulose^[^ ^56^, ^92^ ^]^	Separator (fibrous morphology, thickness N.R.)	Deionized water (pH 5.8, 25 °C)	Depends on the type	Decomposition	Glucosidic chains	Nontoxic
Alginate^[^ ^2^, ^93^ ^]^	Packaging (*⌀* 16 mm and 500 µm thick)	PBS (pH 7.4, 37 °C)	100 wt% in 9 days	Enzymatic hydrolysis by lyases	Oligosaccharides	Biocompatible, purified alginate does not show an adverse reaction when implanted into animals

**Table 3:** Transient behavior of metals, metal oxides, and organic‐based active materials in transient batteries. The degradation rate of metals and metal oxides is usually reported in terms of dissolution rate. Mg(OH)_2_: magnesium hydroxide; Fe(OH)_2_: iron(II) hydroxide; FeO(OH): iron(III) oxide‐hydroxide; H_2_MoO_4_: molybdic acid; Li_3_VO_4_: lithium vanadate; PDCA: pyrrole‐2,3‐dicarboxylic acid; PTCA: pyrrole‐2,3,5‐tricarboxylic acid; TDCA: thiazole‐4,5‐dicarboxylic acid; TTCA: thiazole‐2,4,5‐tricarboxylic acid

Material	Format	Degrading medium	Rate/time	Mechanism	Products	Biological impact
Mg^[^ ^12^, ^49^, ^74^ ^]^	Electroplated^[^ ^49^ ^]^	PBS (pH 7.4, 37 °C), agitated at 50 rpm	2.54 µm h^−1^	Mg + 2H_2_O ⇋ Mg(OH)_2_ + H_2_ Localized corrosion^[^ ^132^ ^]^	Mg(OH)_2_	The degradation product is biocompatible and enhances bone volume in a rabbit animal model
	Foil (50 µm thick)^[^ ^12^, ^74^ ^]^	PBS (pH 7.4, 37 °C)	9–11 days		Mg(OH)_2_	
AZ31 Mg alloy^[^ ^103^ ^]^	Thin film (300 nm thick)	SBF (pH 7.4, 37 °C)	0.02–0.1 µm h^−1^	Mg + 2H_2_O ⇋ Mg(OH)_2_ + H_2_ Localized corrosion^[^ ^132^ ^]^	Mg(OH)_2_	Released Mg^2+^ and Al^3+^ induce mild toxicity at concentrations > 1000 × 10^−6^ m
MZZ alloy^[^ ^103^ ^]^	Extruded rod (8 mm *⌀*)	SBF (pH 7.4, 37 °C)	0.62 µm h^−1^	Mg + 2H_2_O ⇋ Mg(OH)_2_ + H_2_ Localized corrosion^[^ ^132^ ^]^	Mg(OH)_2_	Zn^2+^ ions reduce cell viability of bone‐related cells (MG63 and MC3T3‐E1) in the 10 × 10^−6^–100 × 10^−6^ m range
*β*‐TCP‐coated MZZ alloy^[^ ^103^ ^]^	Extruded rod (8 mm *⌀*)	SBF (pH 7.4, 37 °C)	0.365 µm h^−1^	Mg + 2H_2_O ⇋ Mg(OH)_2_ + H_2_ Localized corrosion^[^ ^132^ ^]^	Mg(OH)_2_	Zn^2+^ ions reduce cell viability of bone‐related cells (MG63 and MC3T3‐E1) in the 10 × 10^−6^–100 × 10^−6^ m range
Fe^[^ ^103^ ^]^	Foil (10 µm thick)	PBS (pH 7.4, 37 °C)	0.0034 µm h^−1^	Fe + 2H_2_O ⇋ Fe(OH)_2_ + H_2_ Localized pitting corrosion^[^ ^133^ ^]^	Fe(OH)_2_, FeO(OH)	Fe^2+^ and Fe^3+^ ions in the 100 × 10^−6^–1000 × 10^−6^ m range show moderate toxicity
Mo^[^ ^134^ ^]^	Foil (10 µm thick)	PBS (pH 7.4, 37 °C)	0.00083 µm h^−1^	2Mo + 2H_2_O + 3O_2_ ⇋ 2H_2_MoO_4_ Good pitting corrosion resistance^[^ ^135^ ^]^	Mixed oxides	Excess Mo (70–2000 mg L^−1^) in soil, water, and air is absorbed by terrestrial and aquatic organisms, leading to chronic toxicity
MoO_3_^[^ ^136^ ^]^	Powder	Aqueous solution (pH 10, 30 °C)	5.556 × 10^−7^ min^−1^	MoO_3_ (s) + 2OH^−^ ⇋ H_2_O + MoO_4_^2−^ Soluble in H_2_O	H_2_MoO_4_	High doses of molybdate ions (>3 × 10^−3^ m) can induce toxicity to cells
MnO_2_^[^ ^136^ ^]^	Powder	Leibovitz's L15 cell culture medium (pH 7; no temperature reported)	≈20% in 72 h	MnO_2_ + 4H^+^ + 2e^−^ ⇋ Mn^2+^ + 2H_2_O Reductive degradation, biosoluble	Mn^2+^	High MnO_2_ doses (100–250 µg mL^−1^) cause lactate dehydrogenase leakage during in vitro toxicity evaluation
V_2_O_5_^[^ ^2^ ^]^	Nanofiber (100 nm *⌀*, 10 µm long)	4 m LiOH aqueous solution (pH 14, 25 °C)	20 min (no mass loss reported)	2LiOH + V_2_O_5_ → 2LiVO_3_ + H_2_O, LiVO_3_ + 2LiOH → Li_3_VO_4_ + H_2_O Dissolved under alkali conditions	Li_3_VO_4_	Bulk V_2_O_5_ is genotoxic
Melanin^[^ ^137^ ^]^	Slabs (100 µm thick, 5 mm *⌀*)	In vivo degradation in Sprague‐Dawley rats (near sciatic/peripheral nerve, 37 °C)	≈8 days	Gross erosion	PDCA, PTCA, TDCA, TTCA	Biocompatible, melanin induces a benign effect on nerve tissue
Quinone redox species, e.g., hydroquinone^[^ ^138^ ^]^	Salt solution (up to 7262 µM)	*Penicillium chrysogenum var. halophenolicum* (fungus), saline medium, 25 °C	75% after 56 h	Fungal degradation	Benzoate (anaerobic), *β*‐ketoadipate (aerobic)	Biodegradable, human carcinogen; slight toxicity for aquatic organisms, not harmful for bacteria and fungi

### 2.1 Packaging and Separator

Thanks to their physicochemical properties, low cost, and ease of processing, polymers are commonly applied as packaging and separator materials in transient batteries. Current batteries mainly rely on petroleum‐derived polymers as separator and electrode binder materials.^[^ ^57^ ^]^ However, natural biopolymers have also gained attention and are now applied in batteries as highly electrolyte‐wettable and thermally resistant separators, as binders in electrodes sustaining highly reversible electrochemical reactions, or as multifunctional membranes inhibiting the loss of electroactive species during cycling.^[^ ^58^ ^]^ These features contribute to an increased ionic conductivity, enhanced battery safety, improved cycling performance, and enhanced battery life span.

**Figure 5:**
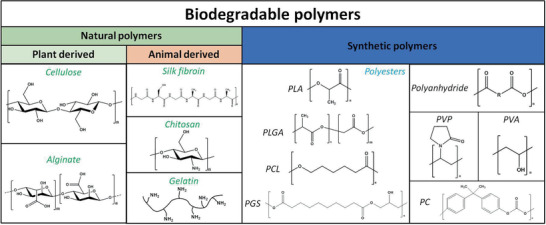
Classification and chemical structure of representative degradable polymers applied as separators and packaging materials in transient batteries according to their source. PLA: polylactide; PLGA: poly(lactic‐*co*‐glycolic acid); PCL: poly(*ε*‐caprolactone); PGS: poly(glycerol sebacate); PVP: polyvinylpyrrolidone; PVA: polyvinyl alcohol; PC: polycarbonate. Gelatin is depicted by a simplified model.

As schematically summarized in **Figure** **5**, two types of degradable polymers can be distinguished depending on the source: naturally derived and synthetic polymers.^[^ ^59^ ^]^ Naturally occurring polymers are further classified into plant‐derived polysaccharides (e.g., cellulose, alginate) and animal‐derived materials (e.g., chitosan, silk). Natural polymers offer excellent properties such as biocompatibility, nontoxicity, easy availability, and affordability.^[^ ^60^ ^]^ Disadvantages of natural polymers include a marked batch‐to‐batch variation due to different sources of origin, a strong immunogenic response associated with their bioactivity, and they require complex and expensive purification procedures.^[^ ^61^ ^]^ Synthetic polymers, on the other hand, display predictable properties, batch‐to‐batch uniformity, and they can be tailored to provide the desired physicomechanical properties for specific applications.^[^ ^62^, ^63^ ^]^

Among synthetic polymers, polyesters, polyanhydrides, and polycarbonates are the most widely applied degradable polymers in transient batteries. Some of the synthetic polymers commonly found in transient devices can be extracted from biomass as well. Obtaining synthetic polymers from biomass can contribute toward sustainability and ultimately toward the circular economy, thus fulfilling one of the goals of transience technology. One of the most prominent polymers in this sense is polylactide (PLA), which can be entirely produced from lactic acid (LA), a product extracted from the fermentation of agricultural products.^[^ ^64^ ^]^

Polymer degradation occurs when the average length of the main chain is reduced through the cleavage of chemical bonds.^[^ ^65^ ^]^ The degradation depends on environmental conditions (pH, temperature) and physicochemical properties of the polymer (crystallinity, molecular weight, etc.). Overall, four main biomaterial degradation mechanisms are found: hydrolytic degradation, enzymatic degradation, oxidative degradation, and physical degradation. Generally, naturally derived polymers are prone to undergo enzymatic or oxidative degradation, whereas synthetic biodegradable polymers are susceptible to hydrolytic degradation.^[^ ^66^ ^]^

Based on triggers such as water, light, temperature, or pH changes, fully transient electronics (full dissolution), and partially transient electronics (partial disintegration) have been reported so far.^[^ ^54^ ^]^ Either way, the packaging is the first material suffering transiency as it shields the battery from the external environment. The transient properties of the packaging are directly related to the morphological, structural, and chemical features of the encapsulation. The thickness of the polymeric packaging, its molecular weight, and crystallinity degree are some of the key characteristics that can be easily modulated to achieve tailored transiency. As a matter of fact, whether surface or bulk erosion occurs is mainly related to the chemical nature of the polymers themselves, e.g., while PLA or polyglycolic acid (PGA) show a bulk degradation process, poly(ortho esters) are inherently surface‐eroding polymers.^[^ ^67^ ^]^ However, the geometry and shape of the polymeric components (thickness, porosity) can influence such degradation mechanism by determining the contact surface area and the prospective water diffusion kinetics into the bulk. Generally, thick films are degraded following a surface erosion mechanism, while a shift from surface to bulk erosion can occur provided the thickness drops below a critical thickness value.^[^ ^68^ ^]^ Moreover, in the case of bulk erosion, the thicker the sample, the faster the degradation as products autocatalyze degradation reactions when accumulated within the interior of the sample. In surface erosion, larger surface‐to‐volume ratios accelerate degradation reactions by exposing additional polymer chains to chain scission reactions. Additionally, larger molecular weights delay degradation kinetics as a result of the less available chain ends to undergo catalytic reactions,^[^ ^69^ ^]^ while polymers having large amorphous regions are more sensitive to hydration and in consequence to hydrolysis, boosting their transiency.

Hereunder, the most commonly found degradation mechanisms of polymers applied either as packaging materials or separators in transient batteries are discussed. The most common thicknesses of polymeric packaging are in the range of 40–150 µm (to effectively protect the battery from surrounding medium), although packaging thicknesses reaching up to 500 µm have also been reported.^[^ ^2^ ^]^ Regarding the separators, thicknesses from 50 to 300 µm are commonly observed in transient batteries as they offer a compromise between resistance against dendrites and ion diffusion resistance.^[^ ^8^, ^70^ ^]^ The reaction kinetics of hydrolytic and enzymatic degradation as well as environmental or biological effects of the released products are also summarized in the next section.

#### 2.1.1 Hydrolytic Degradation

Hydrolytic degradation is the main degradation mechanism of synthetic polymers (polyesters mostly) and is based on a water‐induced random scission of susceptible bonds.^[^ ^61^ ^]^ Different factors can yield hydrolyzable bonds. The formal charge of the reacting carbon markedly affects the reactivity of the polymer toward hydrolysis. During hydrolysis, the oxygen atom of H_2_O attacks the positively charged carbon atoms of the macromolecules via a 2nd order nucleophilic substitution reaction, and therefore chemical groups with a charge value above 0.3 electron charges are hydrolytically active.^[^ ^69^ ^]^ This is of particular relevance in the case of esters, amides, carbonates, carbamates, ureas, anhydrides, and orthoesters, which are vulnerable to hydrolysis as a result of their charge value >0.3. Similarly, conjugated structures influence the reaction kinetics as their presence stabilizes the chemical groups, hindering bond scission. Additionally, steric effects can make hydrolyzable bonds less accessible to cleavage.^[^ ^69^ ^]^

**Figure 6:**
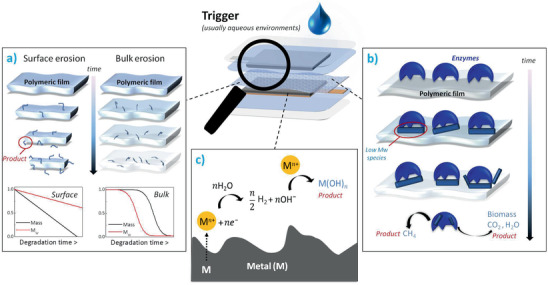
Schematic degradation of different components in transient batteries: a) hydrolytic degradation process of a polymer showing surface versus bulk erosion; bottom insets: plots of the surface and bulk degradation effects on the weight average molecular weight *M*_w_ and remaining mass of polymers versus time, b) enzymatic degradation process of a polymer at different time points, c) degradation of metallic components in transient batteries.

The determination of how the degradation of polymeric materials proceeds and controlling chain‐scission events is important for a rational design of transient batteries. As schematically shown in **Figure** **6**a, polymers undergo two main hydrolytic degradation mechanisms, namely bulk degradation and surface erosion. Understanding the type of degradation process is critical in biomedical applications because bulk degradation implies a rapid and short release of degradation products, where the weight average molecular weight *M*_w_ is markedly reduced. Such *M*_w_ reduction yields soluble chains which can be expelled into the surrounding medium. This degradation process is characterized by a simultaneous *M*_w_ reduction and mass loss throughout the whole specimen. On the contrary, surface erosion consists of a controlled layer‐by‐layer degradation (only at the surface, the bulk remains invariable), resulting in a linear and controlled mass loss and *M*_w_ decreases over time.^[^ ^53^ ^]^ Water diffusion determines whether bulk degradation or surface erosion will occur. If diffusion is faster than the hydrolysis of surface chains, a bulk erosion process takes place. The polymer is thus saturated by the degrading medium (typically an aqueous solution), and a nonlinear mass loss occurs over time. During this process, degradation products (oligomers) containing hydroxyl and carboxylic acid groups are accumulated within the polymer matrix and consequently, the reaction is autocatalyzed.^[^ ^66^ ^]^ Conversely, when the hydrolysis of surface chains is fast, surface erosion occurs. As autocatalytic effects are suppressed due to the unrestricted diffusion of degradation products away from the polymer matrix, this process is generally expressed as a linear loss in mass over time.^[^ ^66^ ^]^ These two mechanisms are not independent and a combination of both may also occur.

Among synthetic polymers, polyesters, wherein repeating units are bonded via ester linkages, show ideal properties to be applied in transient batteries. Their high susceptibility to nucleophilic attack by hydroxide ions make them suitable alternative materials to traditional petroleum‐based nonbiodegradable polymers.^[^ ^69^ ^]^ PLA is the most prominent polyester which has found application in transient batteries as both encapsulating and separator material.^[^ ^33^ ^]^ For instance, a thick PLA film was used as the encasement for an electrochemical cell, together with a PLA spacer, to print the electrode pair and confine the NaCl/poly(*ε*‐caprolactone) (PCL) composite inside the cell. PLA showed good tensile strength and remarkable compatibility with PCL. As shown in Table 2, the hydrolytic degradation of PLA is initiated by the diffusion of water molecules into the matrix, cleaving ester bonds that release lactic acid oligomers with carboxyl and hydroxyl end groups.^[^ ^71^ ^]^ Subsequently, autocatalysis starts due to the increase in acidic conditions. The formed lactic acid oligomers diffuse through the material and dissolve in water. Diffusion is assisted by the plasticization effect of water, which increases the free volume. In this case, degradation happens faster in the bulk of the sample than on the outer layer.^[^ ^71^ ^]^ Amorphous PLA loses 50% of its original weight in 8 days at 70 °C and pH value of 5.4, with alkaline media and elevated temperatures favoring its degradation process. The as‐generated degradation products include LA, CO_2_, and H_2_O, which can be metabolized in the body or can be ejected through urine and breath, making PLA a suitable material for transient batteries aimed at biomedical applications.^[^ ^72^ ^]^ However, it should be taken into account that LA may cause severe inflammation of surrounding tissues due to its low p*K*_a_ value (the logarithmic acid dissociation constant) of 3.08,^[^ ^73^ ^]^ highlighting that a complete understanding of the transiency products is crucial.

As some polymers do not display the mechanical or chemical characteristics required for transient batteries, copolymerization has been pursued as a fruitful strategy to upgrade their functional properties. Poly(lactic‐*co*‐glycolic acid) (PLGA), which is a copolymer of PLA and PGA, is one of the most remarkable examples. PLGA was used as an encapsulation material to develop a fully biodegradable primary magnesium–molybdenum trioxide (Mg–MoO_3_) battery system.^[^ ^12^ ^]^ Using Mg and MoO_3_ as the anode and cathode, respectively, and an alginate‐based hydrogel electrolyte, a battery lifetime of 13 days was achieved, which is larger than most of the reported transient batteries that last for a maximum of 4 days.^[^ ^8^, ^49^, ^74^, ^75^ ^]^ Accordingly, this system seems appropriate for therapeutic stimulation functions that operate for a few weeks. Moreover, full in vivo and in vitro biodegradability was achieved. The degradation kinetics of PLGA films in phosphate‐buffered saline (PBS) solution revealed that as soon as the films were immersed in the liquid environment, water diffused throughout the samples to yield bulk chain scission events.^[^ ^76^ ^]^ As a result, PLGA was degraded into its LA and glycolic acid (GA) units, releasing acidic molecules into the solution which caused a pH decrease.^[^ ^76^ ^]^ These oligomers are eventually broken down to yield CO_2_ and H_2_O. The LA‐to‐GA ratio determined the degradation kinetics of PLGA. With increasing LA content, the degradation kinetics slowed down due to the hydrophobicity of LA and higher glass transition temperature which increased chain stiffness and reduced the susceptibility of ester groups to hydrolysis.^[^ ^68^ ^]^ A PLGA with a 50:50 LA‐to‐GA ratio showed a 20% weight loss after 10 days in PBS (pH 7.4, 37 °C).^[^ ^76^ ^]^ PLGA shows excellent biocompatibility properties due to the easy assimilation and transformation of LA and GA by Krebs cycle.^[^ ^12^ ^]^

Taking advantage of the hydrolytic degradation of PCL without any toxic effects,^[^ ^77^, ^78^ ^]^ a biodegradable magnesium/iron battery with a 5 µm thick PCL layer, which served both as a packaging and a permeable coating to absorb liquid electrolyte, was constructed and tested under in vitro conditions.^[^ ^49^ ^]^ The thickness of the PCL packaging determines the stability and performance of the battery. An optimum thickness between 30 and 50 µm was required to confine the electrolyte without significantly increasing the mass transfer resistance, thus enabling a battery lifetime of more than 24 h. As PCL is a slowly degrading polyester, it is particularly suitable for batteries aimed at longer lifetimes. In fact, in vitro degradation studies performed at 37 °C using PBS and simulated body fluid (SBF) revealed that PCL barely loses 3.3% of its weight after 90 days.

Poly(glycerol sebacate) (PGS) is a simple glycerol‐ester‐based polymer that was applied as a substrate in degradable electronic devices. PGS–cinnamate together with silver nanowires was applied as electrode material of an ingestible current source.^[^ ^11^ ^]^ Both in vivo and in vitro studies indicate that PGS undergoes surface degradation, which avoids a sudden release of degradation products and makes this polymer especially useful for biomedical applications. A PGS implant in the subcutaneous area of Sprague‐Dawley rats degrades fully within 60 days, while only 17.6% of its weight is lost in 60 days in PBS at 37 °C.^[^ ^79^ ^]^ As PGS is composed of naturally occurring glycerol and sebacic acid monomers, the human body can easily metabolize the degradation products. Besides, no catalysts or additives are necessary during PGS synthesis, avoiding possible toxic effects when intended for biomedical applications.^[^ ^80^ ^]^

In addition to polyesters, other classes of synthetic polymers have been applied in transient batteries. Water‐activated primary batteries with a Mg anode and Fe, W, or Mo cathodes were packed using polyanhydride.^[^ ^74^ ^]^ Polyanhydrides have a hydrophobic main chain linked by easily hydrolyzable anhydride groups which undergo degradation through a surface erosion process.^[^ ^81^ ^]^ Polyanhydrides together with PLGA were applied in a primary Mg–MoO_3_ transient battery as coatings capable of being fully degraded within 48 h in moisturized environments.^[^ ^12^ ^]^ Polyanhydrides degrade in vitro and in vivo into their corresponding acids with no biologically adverse effects, demonstrating their high potential for biocompatible energy storage devices.^[^ ^15^ ^]^

Water‐soluble polymers such as polyvinylpyrrolidone (PVP) and polyvinyl alcohol (PVA) are other frequently applied synthetic polymers in transient batteries. A transient battery capable of dissolving in water in solely 10 min was obtained based on a vanadium oxide (V_2_O_5_) cathode, a Li metal anode, a nonwoven PVP nanofiber separator, aluminum and copper current collectors, and a sodium alginate encasement.^[^ ^2^ ^]^ The highly porous structure of the PVP separator enables a rapid dissolution once the aqueous trigger is applied. Similarly, a fully degradable battery composed of a Sn‐doped V_2_O_5_ cathode, a Li metal anode, a PVP separator, and a PVA encapsulation was designed by Wang et al.^[^ ^82^ ^]^ As PVA is highly soluble in water, the authors improved the stability of the battery by coating the PVA encapsulation with a thin polycarbonate layer, which is a water‐resistant polymer that rapidly dissolves when exposed to alkaline media. Therefore, when the battery was exposed to an aqueous potassium hydroxide (KOH) solution, a dissolution time for the whole transient battery of only 8 min was achieved.^[^ ^82^ ^]^ Based on a spray coating process, Fu et al. also applied a waterproof polycarbonate layer onto the outer surfaces of the PVA encapsulation to develop a transient lithium pouch cell capable of withstanding corrosion by the surrounding aqueous environment.^[^ ^3^ ^]^

#### 2.1.2 Enzymatic Degradation

Natural polymers attract increasing interest in the energy storage field due to their low cost, functional properties, good film‐forming ability, thermal stability, biocompatibility, and biodegradability.^[^ ^58^, ^83^ ^]^ Natural polymers are degraded through enzymatic processes which involve an enzyme‐catalyzed scission of the polymer chains. Enzymes are biological catalysts capable of accelerating the reaction kinetics without undergoing any permanent changes.^[^ ^84^ ^]^ As depicted in Figure 6b, the process typically occurs in four steps: 1) diffusion of the enzyme from solution to the polymer surface, 2) enzyme adsorption on the surface of the polymer to create an enzyme–substrate complex, 3) catalysis of the bond cleavage, and 4) diffusion of the soluble degradation products away from the substrate and finally bioassimilation and mineralization. Depending on the environment where the degradation occurs, different types of enzymes are required to catalyze the depolymerization reactions. Hereafter, the enzymatic degradation of naturally derived polymers applied in transient batteries is summarized.

Among animal‐derived biopolymers, silk fibroin (SF, a structural protein obtained from silkworm, insects, or spiders) and chitosan (a linear polysaccharide extracted from crustaceans) have been applied in transient batteries, thanks to their facile processability, mechanical strength, and versatile functionalization, all of which are advantageous in comparison with other animal‐derived biopolymers.^[^ ^85^, ^86^ ^]^ Biopolymers can be combined with an ionic liquid (IL), which is a salt in the liquid state at temperatures below 100 °C, forming a polyelectrolyte.^[^ ^87^ ^]^ A SF–choline nitrate (SF–[Ch][NO_3_]) polyelectrolyte was reported in a fully biodegradable thin‐film Mg battery by Jia et al.^[^ ^8^ ^]^ As a result of the combination of SF with the biocompatible IL, the ion‐conducting membrane was degraded in a buffered protease XIV solution after 24 h with 89% weight loss. The presence of the IL favored the formation of the amorphous structure of SF, allowing a high transiency rate.^[^ ^88^ ^]^ Moreover, the good biocompatibility of SF makes this material an interesting platform to develop transient devices.^[^ ^89^ ^]^

Chitosan, a linear polysaccharide formed by d‐glucosamine and *N*‐acetylglucosamine molecules connected by *β*‐(1→4) linkages, has been applied as a host material to obtain ionically conducting membranes. For example, chitosan was combined with [Ch][NO_3_] to obtain a biocompatible Mg–air battery for implantable applications.^[^ ^32^ ^]^ Chitosan provides good mechanical support and dimensional stability, while [Ch][NO_3_] supplies charge carriers and acts as a plasticizer. As a result, a mechanically flexible and highly ionically conducting material was obtained. Although different enzymes can degrade the *β*‐(1→4) linkages, lysozyme is the most commonly found enzyme for chitosan degradation. However, special care should be paid to the chitosan deacetylation degree (DD) as DDs above 95% are not degradable by lysozymes.^[^ ^90^ ^]^ Gelatin is another biodegradable animal‐derived polymer with huge potential in transient devices. Gelatin is a collagen‐derived polymer and it has been extensively applied in food and pharmaceutical industries because of its good film‐forming properties, low price, nontoxicity, and biodegradable character.^[^ ^91^ ^]^ As a matter of fact, an edible and biodegradable electrochemical power source packaged within a gelatin capsule to facilitate oral delivery was reported.^[^ ^11^ ^]^ When the device is hydrated, the gelatin capsule is dissolved and allows hydration of the electrodes, which are deployed to contact each other and initiate the discharge of the electrochemical sodium ion cell (activated carbon as anode and *λ*‐MnO_2_ as cathode; initial potential of 0.6 V and energy density of 0.3 Wh kg^−1^).

Cellulose and alginate are the most common plant‐derived polymers in transient batteries. Cellulose, which can be extracted from wood or cotton, has been used as a platform material to fabricate porous separators for a LIB capable of undergoing transiency in 30 min (when exposed to water) through a combination of dispersion of insoluble and dissolution of soluble components.^[^ ^56^ ^]^ Cellulose is best degraded when exposed to cellulases, which hydrolyze *β*‐(1→4) glycosidic linkages to obtain glucose molecules.^[^ ^92^ ^]^ Sodium alginate, a polysaccharide that can be extracted from brown algae, was applied as a water‐soluble encapsulating material for a rechargeable lithium‐based transient battery capable of delivering high voltage and capacity.^[^ ^2^ ^]^ Despite its high solubility in water, sodium alginate shows good stability in conventional organic electrolytes, making this material suitable for transient batteries containing organic liquid electrolytes. When exposed to enzymatic environments, lyases cleave sodium alginate chains via a *β*‐elimination mechanism, resulting in biocompatible oligosaccharides.^[^ ^93^ ^]^

### 2.2 Active Materials and Current Collectors

The current collector is a critical component of batteries as it provides mechanical support to the electrode materials (cathode/anode) and collects electrons from them. In conventional batteries, the selection of active materials and current collectors is mainly based on their electrochemical stability and performance. These requirements become more stringent when it comes to transient batteries as these materials should not only show high electrochemical performance but also adequate degradability in a suitable fluidic solution. Moreover, for applications in the human body, these materials should exhibit biocompatibility and generate degradable products with minimum deleterious effects. Regarding nonbiological applications, the materials are expected to show stable performance during their operation period, and then disappear in their surroundings at controlled rates without releasing toxic products.

**Figure 7:**
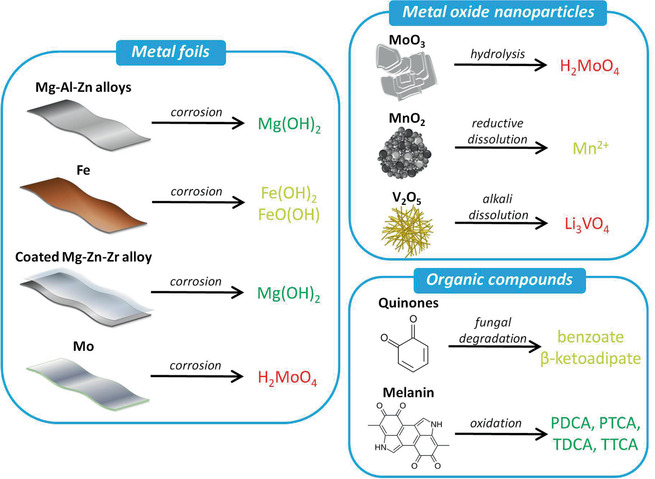
Transiency of active materials used in batteries, classified into metal foils, metal oxide nanoparticles, and organic compounds. Each material is schematically shown according to its physical appearance. The transiency mechanism and released products are summarized following a color code depending on the potential biological or environmental impact (green for negligible; yellow for intermediate; red for large). PDCA: pyrrole‐2,3‐dicarboxylic acid; PTCA: pyrrole‐2,3,5‐tricarboxylic acid; TDCA: thiazole‐4,5‐dicarboxylic acid; TTCA: thiazole‐2,4,5‐tricarboxylic acid. Quinones degrade into benzoate or *β*‐ketoadipate under anaerobic and aerobic conditions, respectively. Mg(OH)_2_, H_2_MoO_4_, Mn^2+^, Li_3_VO_4_, benzoate, *β*‐ketoadipate, PDCA, PTCA, TDCA, and TTCA are water soluble, while Fe(OH)_2_ and FeO(OH) present a poor solubility in water.

Biodegradable metals, metal oxides, and organic‐based materials are the most commonly employed electrode materials for transient batteries designed to power IMDs (see the schematic classification of active materials in **Figure** **7**). Biodegradable metals also serve as current collectors due to their low electronic resistance, biocompatibility, and excellent mechanical properties. These metals corrode gradually in vivo with no harmful effects and completely disappear without any residues.^[^ ^98^ ^]^ Typically used biodegradable metals in transient batteries include Mg, Mg‐based alloys, iron (Fe), molybdenum (Mo), zinc (Zn), and tungsten (W).^[^ ^8^, ^49^, ^74^, ^99^ ^]^ All these metals except W are essential metallic elements for the human body with significant physiological roles. Metal oxides such as MoO_3_ and manganese dioxide (MnO_2_) were applied as cathode materials for biodegradable batteries due to their high solubility in aqueous solution, biocompatibility at controlled levels, and edibility.^[^ ^11^, ^12^ ^]^ Metal oxides have been widely applied as electrode materials in conventional batteries (LIBs, sodium‐ion batteries (NIBs), zinc‐ion batteries, etc.) due to their large theoretical capacities, capacity retention, and cyclability.^[^ ^100^, ^101^ ^]^ Generally, metal oxides such as titanium, manganese, or zinc oxides are chemically and thermally stable, can be obtained through large‐scale manufacturing approaches, are abundant, affordable, and their working voltage and energy density can be tuned by morphology and chemistry design (particle shape, crystal structure, stoichiometry).^[^ ^99^, ^101^, ^102^ ^]^

Gold (Au) nanoparticles as electrode materials offer bioinertness and catalytic properties.^[^ ^8^ ^]^ Alternatively, transient batteries targeting ex vivo applications mostly exploit dissoluble electrodes especially V_2_O_5_, metallic Li, and conducting metals such as copper (Cu) and aluminum (Al) for current collectors.^[^ ^2^, ^3^, ^82^ ^]^ These materials are chosen based on their electrochemical stability in organic electrolytes and fast dissolution behavior in alkaline solution formed by the reaction of Li metal with water. Among nonmetallic electrodes, eumelanins, a subclass of melanin pigments, and quinone redox species, were explored as anode materials for aqueous batteries. These biologically derived materials are well‐known to exhibit excellent in vitro and in vivo biocompatibility along with biodegradability via free radical degradation mechanism.^[^ ^75^ ^]^

A complete understanding of the degradation mechanisms, kinetics, and electrochemical performance of the abovementioned materials is essential for developing more advanced and high performing transient batteries. The degradation behavior of materials depends on a multitude of factors ranging from solution chemistry to the redox environment. Additionally, it is critical to assess the impact of generated products on the immediate surroundings, either within the human body or in natural environments. In the following section, these key points involving the dissolution mechanism, kinetics, and potential biological or environmental impact of the active materials and current collectors proposed for transient batteries will be discussed.

#### 2.2.1 Dissolution Behavior of Metals

Most of the efforts reported to date have been devoted to the development of nonrechargeable (primary) batteries. Therefore, if not stated otherwise, the batteries reported here are intended for single use. The general mode of degradation of biodegradable metals is through a corrosion process. As summarized in Figure 6c and Table 3, when immersed in water/biofluids, metals undergo electrochemical reactions (oxidation) to form metal cations and other reaction products such as oxides, hydroxides, phosphates, and hydrogen gas.^[^ ^103^ ^]^ As a well‐known biodegradable metal, Mg is routinely found in structural implants and is the preferred choice as anode material in transient primary batteries due to its high theoretical specific charge capacity (2200 mAh g^−1^), high physiological tolerance (300 mg per day), biocompatibility, and appreciable negative electrode potential (−2.3 V vs standard hydrogen electrode).^[^ ^49^, ^99^ ^]^ Mg degrades in aqueous solution to produce hydrogen gas and magnesium hydroxide (Mg(OH)_2_), a biocompatible corrosion product shown to enhance bone growth in vivo.^[^ ^104^ ^]^ The degradation behavior of Mg is influenced by a variety of factors including the composition of degrading media, temperature, and the shape/form in which Mg is conformed in the batteries. Different shapes of Mg used in transient batteries include thick Mg films micropatterned by subtractive etching of Mg foil, electrodeposited Mg microstructures from organic solvents, and foil format. They all differ from each other in terms of crystal orientation, electrical resistivity, surface morphology and grain size that eventually dictate their degradation behavior. Tsang et al. studied the degradation behavior of electroplated Mg in PBS (pH 7.4, 37 °C) and revealed its lower corrosion resistance in comparison to commercial Mg foil, most likely due to its larger grain size and high surface roughness.^[^ ^105^ ^]^ The same group used electroplated Mg as anode for PCL‐encapsulated Mg–Fe biodegradable batteries and demonstrated its complete dissolution within 20 days in PBS solution (pH 7.4, 37 °C), agitated at 50 rpm to mimic body conditions.^[^ ^49^ ^]^ Thick foil format of Mg as anode and current collector was utilized in fully transient Mg–Mo and Mg–MoO_3_ battery systems.^[^ ^12^ ^]^ In Mg–Mo primary batteries, the dissolution of Mg and Mo foils together with a polyanhydride encasing took place slowly in PBS solution at 37 °C for the initial 11 days and then completely disappeared in the next 8 days as the temperature of the solution was increased to 85 °C. Conversely, the degradation of Mg–MoO_3_ proceeded faster as the Mg foil, the sodium alginate hydrogel, and the MoO_3_–PLGA layer completely disappeared in PBS solution (pH 7.4, 37 °C) within 9 days. These different transiency times are related to the different dissolution rates of Mg and Mo foils, reported to be 1–10 and 0.02 µm per day, respectively.

Despite the attractive properties of Mg as anode, its rapid corrosion in aqueous solution and high self‐discharge rates have limited its application. To obtain longer degradation times and better performance for transient Mg‐based batteries, alloying of Mg with biocompatible metals and surface coating methods were developed. AZ31 Mg alloy containing 3% Al and 1% Zn by weight was used as anode material in primary Mg batteries, increasing battery lifetime by 6 times compared to pure Mg.^[^ ^106^ ^]^ A dissolution rate of 0.05–0.5 µm h^−1^ was observed for a Mg thin film in SBFs (pH 7.4, 37 °C), while a AZ31 thin film dissolved at a rate of 0.02–0.1 µm h^−1^ due to its improved corrosion resistance.^[^ ^107^ ^]^ Khan et al. developed a Mg–Zn anode system for transient Mg primary batteries via combinatorial magnetron cosputtering.^[^ ^99^ ^]^ The rationale behind their study was to identify the optimum combination in the Mg–Zn system that would provide higher electrochemical performance and longer lifetime. The corrosion resistance of Mg was found to be improved with increasing Zn concentration; however, no degradation experiments were reported in the paper. A new biodegradable anode consisting of Mg–Zn (3 wt%)–Zr (0.8 wt%) (MZZ) alloy coated with biocompatible *β*‐tricalcium phosphate nanorods (*β*‐TCP) was also developed.^[^ ^108^ ^]^ The degradation rate of MZZ and *β*‐TCP‐coated MZZ alloy was monitored by measuring the weight loss in both samples after long‐term immersion in SBF at 37 °C. The average corrosion rate of *β*‐TCP‐coated MZZ alloy (0.365 µm h^−1^) was found to be slower than that of MZZ alloy (0.62 µm h^−1^), thus confirming the protective nature of the coating. Structural analysis showed the presence of many corrosion pits on the surface of MZZ alloy, while *β*‐TCP‐coated MZZ alloy maintained its structural integrity without any dramatic change. Tsang et al. minimized the parasitic corrosion of Mg anode by passivating its surface with biodegradable polymers, i.e., PCL and PGS for microelectromechanical‐system‐enabled biodegradable batteries.^[^ ^109^ ^]^ However, no dissolution experiments were conducted to show the effect of these polymers on the overall dissolution behavior of Mg under physiological conditions.

Among the cathode materials for transient batteries, Fe is most widely explored due to its advantageous mechanical and electrochemical properties. Degradation of Fe in biofluids and distilled water occurs very slowly and in a nonuniform manner due to the formation of insoluble corrosion products (oxides) that accumulate on the surface, preventing further corrosion.^[^ ^107^ ^]^ The dissolution rate of Fe foil in PBS solution (pH 7.4, 37 °C) is reported to be 0.0034 µm h^−1^, which is much slower than the corresponding thin film in Hank's solution (pH values between 5 and 8).^[^ ^107^, ^110^ ^]^ The difference in dissolution rates of metal foil and thin film is attributed to their morphological differences and different compositions of the two solutions. Preliminary in vivo tests in the native descending aorta of pigs (12 months old, mean weight of 23.1 kg) demonstrated excellent biocompatibility of pure Fe in the form of stents with no significant neointimal proliferation, no pronounced inflammatory response, and no organ toxicity.^[^ ^111^ ^]^ Like Fe, Mo foil also has a slower dissolution rate of 0.00083 µm h^−1^ in PBS solution (pH 7.4, 37 °C).^[^ ^110^ ^]^ But unlike iron, the anodic oxidation of Mo in nearly neutral electrolytes yields a soluble product, mainly a mixed‐valence oxide containing Mo(IV), Mo(V), and Mo(VI).^[^ ^112^ ^]^ The exact ratio between different valence states depends on the pH of the degradation medium, and the solubility of this mixed‐valence oxide determines the overall degradation kinetics of Mo. Au nanoparticles (NPs) deposited on biodegradable silk film were applied as a bioinert catalyst toward oxygen reduction reaction for an encapsulated Mg–air battery.^[^ ^8^ ^]^ The biodegradation process of the battery was conducted in buffered protease solution at 37 °C wherein Au NPs physically fragmented in the solution due to the dissolution of the supporting substrate. These NPs were reported to be biocompatible and under optimized enzyme treatment, can be eliminated from the body through renal excretion, phagocytosis, and/or endocytosis.

#### 2.2.2 Dissolution Behavior of Metal Oxides

MoO_3_ is a layered material that is being explored for numerous applications including medical devices, lithium‐ion battery cathodes, chemotherapy agents, and heterogeneous catalysts.^[^ ^113^ ^]^ Huang et al. first reported the potential of MoO_3_ as the cathode for a fully biodegradable primary Mg–MoO_3_ battery.^[^ ^12^ ^]^ A thick slurry of MoO_3_ powder mixed with a biodegradable polymer, PLGA, was cast on top of a Mo foil to obtain a well‐connected 3D network structure. This 3D network structure promoted battery performance due to the increase in effective surface area as well as increased conductivity of the MoO_3_ layer. The transiency of the entire battery was observed in PBS solution at 37 °C. The dissolution behavior of MoO_3_ was found to be controlled by the encapsulation layers, representing an advantage to obtain a desired release rate of Mo in the degradation medium. Being able to control the molybdenum concentration in the solution/electrolyte is essential to achieve maximum cell viability. A concentration of 7 mol% MoO_3_ is compatible with human immortalized keratinocyte (HaCaT) cell line, while higher concentrations showed slightly reduced cell viability.^[^ ^114^ ^]^ The in vitro results indicated that the MoO_3_/PLGA film did not present any toxic effects on L‐929 mouse fibroblast cells and exhibited excellent biocompatibility. The presence of MoO_3_ indeed resulted in increased growth ability of the L‐929 cells. The understanding of the dissolution kinetics of MoO_3_ is critical in determining its fate in the environment and within the human body. The dissolution of MoO_3_ is a slow hydrolysis process, resulting in the formation of molybdate anions as the dominant degradation product following the reaction as shown in Equation (1)^[^ ^115^ ^]^

(1) $$\mathrm{Mo}O_{3}\left(s\right)+H_{2}O⇄2H^{+}+\mathrm{Mo}{O_{4}}^{2-}$$

A very slow dissolution rate of 5.556 × 10^−7^ min^−1^ for MoO_3_ powder in an aqueous solution (pH = 10) was reported.^[^ ^116^ ^]^ An increase in dissolution rate was observed with increasing pH (maximum at pH 9.25 ± 0.2) and temperature (0.0001 min^−1^ at 0 °C compared to 1.09 min^−1^ at 40 °C).^[^ ^116^, ^117^ ^]^ Below pH 2, MoO_3_ is shown to be stable against hydrolysis.^[^ ^118^ ^]^ Recent work demonstrated the dissolution kinetics of MoO_3_ nanoribbons in six different degrading media including Nanopure water (pH 7.0), U.S. Environmental Protection Agency moderately hard water (pH 7.8), phosphate‐buffered saline (pH 7.4), Roswell Park Memorial Institute Medium (RPMI, pH 7.4), simulated lung fluid (SLF, pH 7.5), and phagolysosomal simulant fluid (PSF, pH 4.5).^[^ ^119^ ^]^ The nanoribbons showed complete dissolution in buffered media, i.e., RPMI, PBS, and SLF in which most of the dissolution generated H^+^ ions (see Equation (1)) were neutralized to keep the pH constant, thus shifting the equilibrium to the right favoring faster dissolution. As expected, very little dissolution of MoO_3_ nanoribbons was observed in acidic PSF solution and unbuffered Nanopure water. In vitro toxicity assessment of the nanoribbons demonstrated their rapid dissolution in cell culture and did not prompt any adverse effects for concentrations up to 100 µg mL^−1^. By contrast, exposure to the same dosage of MoO_3_ nanoparticles caused a significant decrease in cell survival after 24 h due to their higher biodurability.

Another interesting metal oxide that has found its way into transient sodium‐ion batteries is MnO_2_. MnO_2_ has many industrial applications in areas such as wastewater treatment, alkaline/Li‐ion batteries, catalysis, sensors, and supercapacitors.^[^ ^120^ ^]^ MnO_2_ exists in different structural polymorphic forms that are categorized into chain‐like tunnel structures, layered, and 3D spinel structures. Among them, *λ*‐MnO_2_ spinel structure with 3D interconnected channels is an ideal cathode candidate for high rate electrochemical cells due to the facile diffusion of ions.^[^ ^121^ ^]^ Kim et al. demonstrated a self‐deployable and edible sodium ion electrochemical cell consisting of activated carbon as anode and *λ*‐MnO_2_ as the cathode.^[^ ^11^ ^]^ The fabrication of the edible battery was achieved through physical lamination of anode and cathode pairs with a conductive PGS–cinnamate/Ag nanowire composite. The amount of Ag nanowires acting as current collector determines the edibility and toxicity of the battery. The amount of Ag in the battery was ≈84.48 µg (3.2 × 24 mm^2^ area), which is within the tolerance limit of the human body (70–88 µg per day).^[^ ^122^ ^]^ Ag nanowires eventually oxidize, corrode, and resorb by the body without any in vitro toxicity. 8 mg of MnO_2_ was used for optimum performance, while the recommended daily allowance of MnO_2_ is 11 mg for adults. Higher doses (100–250 µg mL^−1^) of MnO_2_ particles (size 1–2 µm) have elicited adverse responses such as lactate dehydrogenase leakage during in vitro toxicity evaluation in rat liver cells.^[^ ^123^ ^]^ Regarding the dissolution behavior, MnO_2_ is highly insoluble in water, undergoing reductive dissolution with the release of soluble Mn^2+^ ions in the presence of electron donors. The reductive dissolution of MnO_2_ is a surface‐controlled process and the reduction rate shows strong pH dependence with increasing solubility under acidic conditions.^[^ ^124^ ^]^ Chen et al. demonstrated a unique breakup nature of MnO_2_ nanosheets under mildly acidic conditions and employed this dissolution behavior for drug delivery and ultrasensitive pH‐responsive magnetic resonance imaging applications.^[^ ^125^ ^]^ The dissolution rates of Mn oxides also show a positive correlation with specific surface area and reduction potential.^[^ ^126^ ^]^ In environmental settings, Mn oxides readily undergo cation exchange reactions and are thus applied for adsorptive removal of heavy metal pollutants.^[^ ^127^ ^]^ This property of Mn oxides can be utilized in transient batteries for environmental resorption as the release of these oxides at controlled rates during the degradation process would benefit the environment in the remediation of contaminants in soil and water treatment applications.

Fu et al. reported the first rechargeable transient battery based on mature Li‐ion technology as an extension of transience technology to more advanced batteries.^[^ ^2^ ^]^ The transient rechargeable LIB consisted of V_2_O_5_ as cathode and Li metal as anode along with a biodegradable separator and thin films of Cu and Al deposited onto a sodium alginate substrate as current collectors. V_2_O_5_ is a layered material that has attracted great attention as cathode material for LIBs due to its high theoretical capacity of 294 mAh g^−1^, low price, and abundant sources.^[^ ^3^, ^128^ ^]^ Its choice as the cathode material is mainly ascribed to its dissolution behavior in alkali solution as given by the following reactions in lithium hydroxide (LiOH) solution^[^ ^129^ ^]^

(2) $$2\mathrm{LiOH}+V_{2}O_{5}\to 2{\mathrm{LiVO}}_{3}+H_{2}O$$

(3) $$\mathrm{LiV}O_{3}+2\mathrm{LiOH}\to Li_{3}VO_{4}+H_{2}O$$

In alkaline solution, vanadium exists predominantly in +5 oxidation state, whereas at acidic pH, +4 valence state as vanadyl cations (VO^2+^ and VO(OH)^+^) is favored.^[^ ^129^ ^]^ During the battery dissolution process, basic pH was attained after Li metal, serving as anode for transient LIBs, reacted with water to form LiOH solution. This basic environment also triggered the dissolution of the Al current collector to produce LiAl(OH)_4_ as the degradation product. Cu metal did not dissolve at high pH values, but it was found to disintegrate into smaller pieces due to the dissolution of its supporting substrate, sodium alginate, in water. Although this battery presented a novel degradation mechanism, there are several possibilities to further improve the electrochemical performance. To achieve high areal energy density, a transient LIB was fabricated using LiAl alloy as anode, and an origami‐inspired high‐capacity V_2_O_5_ as cathode.^[^ ^3^ ^]^ Just like Li metal, LiAl alloy also exhibited fast dissolution behavior in 1.5 m KOH solution with a transience time of only 2 min. The origami‐inspired cathode not only provided high areal energy density due to its folded design but also facilitated an increased rate of transience. Further improvement in the electrochemical performance of pure V_2_O_5_‐cathode‐based transient batteries was achieved by doping tin into V_2_O_5_ nanofibers, forming a porous freestanding electrode with a 3D network.^[^ ^82^ ^]^ The Sn‐doped cathode offered a high areal capacity of ≈2 mAh cm^−2^ and upon immersion into concentrated alkali stimuli, V_2_O_5_ fully dissolved into a soluble salt, and the Sn ions formed water‐soluble SnO_3_^2−^. Although V_2_O_5_ is extensively found in transient secondary batteries, it is worth mentioning that many biological response studies demonstrated notable toxicity of this oxide. As a matter of fact, micrometer‐sized V_2_O_5_ was included in the Environmental Protection Agency (EPA) “P‐list” of acutely hazardous chemicals.^[^ ^127^ ^]^ Bulk V_2_O_5_ has been reported to be genotoxic,^[^ ^130^ ^]^ destroyed liver architecture in male guinea pigs, and occupational exposure of workers to vanadium oxide resulted in rhinitis, bronchitis, and pneumonitis.^[^ ^131^ ^]^ The unregulated release of such oxides into the environment during the degradation process of batteries could lead to toxic effects. More recently, a transient primary LIB was developed using biodegradable PVA, cellulose, and active materials such as lithium cobalt oxide (LiCoO_2_) and lithium titanate (Li_4_Ti_5_O_12_).^[^ ^56^ ^]^ The transient behavior of this battery is based on a very interesting approach using chemical dissolution of soluble components such as PVA, cellulose and physical redispersion of insoluble materials, i.e., active materials and carbon black. The detailed degradation behavior of these polymers has been explained in the previous section.

#### 2.2.3 Degradation Behavior of Organic Electrodes

As a promising alternative to conventional inorganic materials, organic compounds have been studied as electrode materials for biodegradable energy storage devices because of their intrinsic advantages like easy fabrication, mechanical flexibility, structural diversity, and acceptable theoretical capacity.^[^ ^75^ ^]^ Melanin pigments were applied as organic electrodes for water‐activated NIBs that can power next‐generation biodegradable electronics.^[^ ^75^ ^]^ Melanins are a broad class of biological pigments found in hair, skin, eyes, and inner ear of different organisms. Melanins consist of two classes of compounds out of which eumelanins (pheomelanins being the other one), the insoluble dark‐brown pigments, were tested in aqueous NIBs due to their unique physical and chemical properties including reversible cation binding abilities. The full cell was fabricated by pairing eumelanin anode with the *λ*‐MnO_2_ cathode and this full cell provided a lifetime of 5 h when operated at a discharge current of 10 µA, much longer than the conventional batteries used in ingestible devices. Although no degradation studies were reported in this work, other literature studies provide information on biocompatibility and biodegradation behavior of melanins. In vitro and in vivo biocompatibility of thin melanin films by examining Schwann cell attachment and growth, as well as neurite extension in PC12 cells, were delineated by Bettinger et al.^[^ ^137^ ^]^ The results pointed to enhanced Schwann cell growth and neurite extension in PC12 cells by melanin thin films, thus confirming its potential as a biodegradable material. Moreover, melanin implants placed near peripheral nerve tissue in Sprague‐Dawley rats were observed to be degradable in vivo with only small fragments remaining after 8 weeks. Chemical degradation of melanin pigments by oxidation with permanganate or hydrogen peroxide, breakdown with hydrogen iodide, and ultraviolet‐induced photodegradation have also been thoroughly studied.^[^ ^139^ ^]^

Quinones, cyclic compounds containing two carbonyl groups in an unsaturated six‐membered ring structure, can be potentially used as electrodes in degradable redox flow batteries, thanks to their good solubility, suitable redox potential, scalability, biodegradability, and low cost.^[^ ^138^ ^]^ However, it should be taken into account that in spite of their biodegradability, degradation products from quinones show acute toxic response in aquatic organisms and can cause cancer in humans.

## 3 Full Cell and Electrochemical Performance

**Figure 8:**
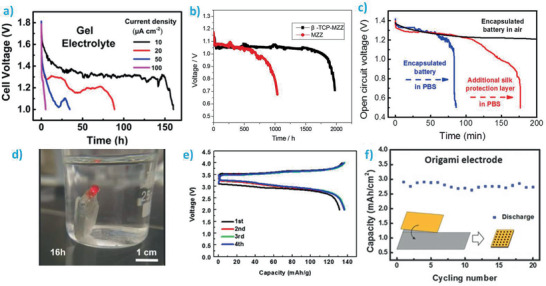
Electrochemical performance of primary transient batteries: a) discharge curves of the Mg–air battery with a chitosan–choline nitrate GPE at different discharge current densities. Reproduced with permission.^[^ ^32^ ^]^ Copyright 2014, American Chemical Society. b) Discharge curves of *β*‐TCP–MZZ and MZZ alloy anode for transient Mg‐based batteries at 100 µA cm^−2^. Reproduced with permission.^[^ ^108^ ^]^ Copyright 2018, Springer Nature. c) Discharge curves of a Mg primary battery with a silk fibroin–choline nitrate GPE and with an additional layer of crystallized silk on top of the encapsulation. Reproduced with permission.^[^ ^8^ ^]^ Copyright 2017, American Chemical Society. d) A red LED powered by Mg–MoO_3_ battery in phosphate‐buffered saline for over 16 h. Reproduced with permission.^[^ ^12^ ^]^ Copyright 2018, Wiley‐VCH. Electrochemical performance of secondary transient batteries: e) Charge–discharge curves at a current density of 100 mA g^−1^ of a transient LIB battery composed of a metallic Li anode and V_2_O_5_ cathode. Reproduced with permission.^[^ ^2^ ^]^ Copyright 2015, American Chemical Society.f) Cycling performance of an origami V_2_O_5_‐cathode‐based LIB. Reproduced with permission.^[^ ^3^ ^]^ Copyright 2016, Wiley‐VCH.

In addition to the degradation mechanism and kinetics, the ability to store energy is of paramount relevance for transient batteries to reach commercialization stage. In this section, we summarize the electrochemical performance of primary and secondary transient batteries (see **Table** **4** and **Figure** **8**). A primary Mg–Mo battery with a specific capacity of 276 mAh g^−1^, open‐circuit voltage (*V*_OC_) of 0.4–0.7 V, and a lifetime of 24 h (limited by the depletion of the active Mg) was reported in PBS liquid electrolyte.^[^ ^74^ ^]^ The low output voltage delivered by the battery was increased by connecting the cells in series to obtain a stable voltage of 1.5–1.6 V for up to 6 h, which is enough to power a light‐emitting diode (LED) and a wireless radio circuit. To extend battery lifetime and improve *V*_OC_, Jia et al. replaced the liquid electrolyte with a chitosan–choline nitrate gel polymer electrolyte (GPE).^[^ ^32^ ^]^ As shown in Figure 8a, the 300 µm thick biobattery demonstrated a *V*_OC_ of 1.33 V (middle point of the discharge curve) for 160 h when cycled at a current density of 10 µA cm^−2^, which is 40 mV above the *V*_OC_ of the batteries with the liquid electrolyte. However, at higher current density, the obtained voltage was 60 mV lower than that of liquid electrolyte due to slower ion mobility inside the GPE. Nonetheless, the biocompatible ionic liquid–biopolymer electrolyte enabled a volumetric power density of 3.9 W L^−1^ for the Mg–air battery, which is adequate to power certain IMDs such as pacemakers or biomonitoring systems.

**Table 4:** Summary of the electrochemical performance of transient batteries including working voltage, specific capacity, lifetime (measured in hours for primary batteries and number of cycles for secondary batteries), Coulombic efficiency, and applied current density. N.R.: not reported; energy density: battery nominal voltage (V) × battery capacity (Ah); power density: discharge current (mA) × battery nominal voltage (V)

	Battery type	Working voltage [V]	Specific capacity [mAh g^−1^]	Energy [μWh]	Power [μW]	Lifetime, Coulombic efficiency [h per cycles, %]	Current density [µA cm^−2^]
Primary	Mg–Mo^[^ ^74^ ^]^	0.6	276	1440	60^a)^	24 h, N.R.	100
	AZ31–air^[^ ^32^ ^]^	1.33	N.R.	2160^b)^	118	160 h, N.R.	10
	Mg–Fe (PCL)^[^ ^49^ ^]^	0.70	1060	3055	30	99 h, 48%	230
	Mg–Fe (PCL–NaCl)^[^ ^33^ ^]^	0.45	N.R.	N.R.	20.25^c)^	24 h, N.R.	100
	Mg–Fe (PCL, PGS)^[^ ^109^ ^]^	1.05 (nominal)^d)^	N.R. (0.667 mAh)	694 μWh	26.2	26.7 h, 12%	25 µA
	MZZ–air^[^ ^108^ ^]^	1.08	N.R.	N.R.	61.07^e)^	1800 h, N.R.	50
	AZ31–air (Au–SF)^[^ ^8^ ^]^	1.03	N.R. (2.2 mAh cm^−2^)	2269	34.24	61 h, 27%	5
	Mg–MoO_3_^[^ ^12^ ^]^	1.5 (initial), 0.6 (final)	N.R.	1971 μWh cm^−2^, 50 h; 6475 μWh cm^−2^, 250 h	N.R. (270 μW cm^−2^ at 300 µA cm^−2^)	50 h@1.5 V, 250 h@0.6 V, N.R.	25
	Na(c)–*λ*‐MnO_2_^[^ ^75^ ^]^	1.03 (initial), 0.43 (average)	16.1 ± 0.8	N.R.	N.R. (4.3 μW)	5 h, N.R.	10 µA
	Na(AC)–*λ*‐MnO_2_^[^ ^11^ ^]^	0.66 (initial), 0.31 (average)	9.95 ± 0.5	0.9 (0.3 Wh kg^−1^)	N.R.	3 h, N.R.	10 µA
	Quinone‐based flow battery^[^ ^141^ ^]^	0.75 ± 0.05 (open‐circuit voltage)	N.R. (0.42 mAh)	N.R. (32 Wh kg^−1^)	1700	0.25–1.42 h (@2000 Ω), 13.3%	18.9 × 10^3^
	Paper‐based redox flow battery^[^ ^142^ ^]^	0.75 ± 0.05 (open‐circuit voltage)	N.R. 4.6 Ah L^−1^ cm^−2^	N.R. 3.1 Wh L^−1^ cm^−2^	710	≤0.42 h, 98%	–
Secondary	Li–V_2_O_5_^[^ ^2^ ^]^	2.80	136.5	290	N.R.	4 cycles, nearly 100%	100 mA g^−1^
	LiAl–V_2_O_5_^[^ ^3^ ^]^	>2.00	187 (3 mAh cm^−2^)	N.R.	N.R.	20 cycles, nearly 100%	2.000
	Sn‐doped Li–V_2_O_5_^[^ ^82^ ^]^	>2.00	168 (3 mAh cm^−2^)	N.R.	N.R.	200 cycles, nearly 100%	300
	LTO–LCO^[^ ^56^ ^]^	2.60 (initial), 1.0 (final)	2.27	N.R.	N.R.	Few cycles (N.R.), 12.5%	20

^a)^ Active area = 1 cm^2^, power = 0.1 mA cm^−2^ × 1 cm^2^ × 0.6 V = 60 μW

^b)^ Battery volume = 300 mm^3^, energy density = 72 Wh L^−1^ × 300 mm^3^ = 2160 μWh

^c)^ Mg area = 0.45 cm^2^, power = 0.1 mA cm^−2^ × 0.45 cm^2^ × 0.45 V = 20.25 μW

^d)^ Nominal voltage = power/discharge current = 26.2 μW × 25 µA^−1^ = 1.048 V

^e)^ Anode area = *⌀* 12 mm, power = 50 µA cm^−2^ × (*π* × 0.6 × 0.6) cm^2^ × 1.08 V = 61.072 μW.

To address the parasitic corrosion of the Mg anode in the presence of liquid electrolytes, which decreases the capacity and energy of the batteries, Tsang et al. protected the electroplated Mg surface with biodegradable polymers like PCL and PGS.^[^ ^49^, ^109^ ^]^ The corrosion resistance of Mg improved with the polymeric coatings, exhibiting corrosion potentials of −1.292 and −1.165 V versus standard hydrogen electrode for PCL and PGS, respectively (compared to −1.320 V for the bare Mg). As a result, the extent of hydrogen evolution originating from the reduction of water upon corrosion is reduced.^[^ ^140^ ^]^ Further, the PCL dip‐coated Mg half‐cells showed a 70% increase in specific capacity at 280 µA cm^−2^ in comparison to the noncoated samples, delivering a maximum specific capacity of 930 mAh g^−1^ at 330 µA cm^−2^. For full cells, the PCL‐coated Mg–Fe batteries managed to achieve a stable discharge voltage at current densities as high as 400 µA cm^−2^ and featured a high energy density of 694 Wh kg^−1^, which is two orders of magnitude higher than that for Mg–Mo batteries. As both thickness and permeability of polymeric coatings influence the mass transfer resistance, the effect of varying the thickness of PCL and PGS on the electrochemical performance of the batteries was studied. The PGS‐coated batteries achieved longer discharge lifetimes than uncoated ones, showing the highest capacity and CE of 0.7 mA h^−1^ and 13.5%, respectively (film thickness of 10 µm). As PGS thickness increases, lower average potential and less stable discharge profiles were obtained due to the increased charge transfer resistance and accumulation of reaction products at the PGS–Mg interface. PCL‐coated batteries showed similar thickness dependence on the battery performance. In addition to the encapsulation layer, a solid electrolyte for Mg–Fe batteries comprising PCL and NaCl was fabricated to maintain the stable electrochemical environment inside the cell, making the battery cell immune to the continuously changing surrounding environment.^[^ ^33^ ^]^ Operating voltages of 0.45 and 0.95 V were obtained for discharge rates of 100 and 12.5 µA cm^−2^, respectively. In another work, a Mg–Fe battery featuring electroplated Mg as anode exhibited a capacity and power of 1.2 mAh and 36 μW at a current of 55 µA, respectively.^[^ ^109^ ^]^ A similar surface coating strategy was pursued with *β*‐tricalcium phosphate nanorods on a biodegradable Mg alloy (MZZ).^[^ ^108^ ^]^ At a current density of 100 µA cm^−2^, the battery with *β*‐TCP–MZZ alloy showed a plateau voltage of 1.05 V for 1800 h in comparison to only 625 h for the noncoated MZZ alloy (Figure 8b). When the current density was increased to 200 µA cm^−2^, the battery sustained a *V*_OC_ of 1.01 V for only 600 h, probably due to the corrosion of *β*‐TCP–MZZ alloy anode during the operation.

Jia et al. demonstrated a silk‐based compact Mg battery using an anode composed of AZ31 Mg alloy, Au NPs deposited onto a crystallized silk film as a cathode, and SF–choline nitrate as a polymer electrolyte.^[^ ^8^ ^]^ The battery showed *V*_OC_ values in the range of 1.45–1.58 V, which dropped immediately when a discharge current was applied. The advantage of using Au in combination with silk as cathode was confirmed by the high plateau voltage and longer lifetimes obtained in comparison with stainless steel mesh cathode (0.86 V and 61 h vs 0.75 V and 9 h, respectively). The full battery delivered a capacity of 2.2 mAh cm^−2^ with a plateau voltage of 1.03 V at a current density of 5 µA cm^−2^. Interestingly, as indicated by the discharge curves in Figure 8c, the battery lifetime under physiological conditions was extended from 64 to 109 min by adding an extra layer of crystallized silk on top of the encapsulated battery.

A fully biodegradable primary Mg–MoO_3_ battery with a *V*_OC_ of 1.6 V, battery lifetime of 50 h delivering 1.5 V (0.6 V for 250 h), and areal energy density of 6.5 mWh cm^−2^ was developed according to the requirements for implantable electronics.^[^ ^12^ ^]^ The output voltage achieved for a single Mg–MoO_3_ cell could power a LED in PBS solution for up to 16 h (Figure 8d) and could drive the amplifier of a low power electrocardiogram signal detector. The thickness and structure of the MoO_3_ electrode and the usage of hydrogel electrolyte as replacement of PBS liquid electrolyte further influenced the electrochemical performance.

A biocompatible NIB based on organic electrodes, i.e., melanin‐based anode together with *λ*‐MnO_2_ cathode, showed a *V*_OC_ of 1.03 V and a specific capacity of 16.1 mAh g^−1^.^[^ ^75^ ^]^ The full cell lasted for 5 h when operated at discharge rates of 10 µA. Unfortunately, the battery suffered from relatively low energy density compared to inorganic electrode materials. The substitution of the melanin‐based anode with activated carbon was also pursued, yielding potentials up to 0.6 V and currents in the range of 5–20 µA.^[^ ^11^ ^]^ Likewise, quinones were applied as electrodes to develop all‐organic and biodegradable redox flow batteries. The quinone‐based redox flow battery reported by Esquivel et al. delivered an open‐circuit voltage of 0.75 ± 0.05 V, able to be increased up to 3.0 ± 0.2 V by stacking four cells in series (which is enough to cover the needs of portable electronic devices).^[^ ^141^ ^]^ Such a primary battery was able to power a water‐monitoring device, although it suffered from a low Coulombic efficiency of 13.3%. The same quinone chemistry has been recently applied in a paper‐based flow battery. Efficiencies as high as 98% were achieved by tuning the size of electrode and flow rate of the battery.^[^ ^142^ ^]^ The primary battery featured a lifetime of about 30 min and a cell energy density of 3.1 Wh L^−1^ cm^−2^ for an electrode length of 20 mm.

Regarding secondary batteries, the first transient LIB was shown in 2015 by Fu et al. through a combination of cut‐and‐stack and shadow mask metal deposition techniques.^[^ ^2^ ^]^ The battery presented a working voltage of 2.8 V and was able to deliver an energy of 0.29 mWh (energy density of 480 Wh kg^−1^). As shown in Figure 8e, the battery could withstand four charge–discharge cycles with a discharge capacity of 131.3 mAh g^−1^ and CE of 99%. A step forward in this direction came from the development of an origami V_2_O_5_‐cathode‐based LIB, which showed a stable cycling performance for up to 20 cycles (Figure 8f).^[^ ^3^ ^]^ The transient battery offered a high areal capacity of 3 mAh cm^−2^ (specific capacity of 187 mAh g^−1^) and a high working voltage above 2.0 V, comparable to conventional LIBs. To further enhance the battery performance, metal ion doping was used to synthesize a Sn‐doped V_2_O_5_ cathode.^[^ ^82^ ^]^ This battery provided 0.27 mAh cm^−2^ capacity at a current density as high as 17.76 mA cm^−2^. The transient battery with the Sn‐doped V_2_O_5_ cathode had a capacity of 2 mAh cm^−2^ at an areal current density of 0.3 mA cm^−2^ compared to 1.2 mAh cm^−2^ for the pure V_2_O_5_ cathode. The high performance of the doped cathode was ascribed to its better electronic conductivity and electrochemical reversibility. Finally, a transient LIB battery based on a LiCoO_2_ (LCO) cathode and a Li_4_Ti_5_O_12_ (LTO) anode generated a total specific capacity of 2.27 mAh g^−1^ and a CE of 12.5% when discharged at 20 µA cm^−2^.^[^ ^56^ ^]^ The battery was able to power a 1 V calculator for ≈15 min. However, the transient LIB presented a relatively poor electrochemical performance due to nonuniform interfaces and poor connections between the battery components.

The power provided by transient batteries reported so far is enough to support ultralow power IMDs and biosensors, which require output voltages from 0.3 to 1.5 V.^[^ ^143^ ^]^ For example, batteries delivering 30–100 μW are sufficient for pacemakers and cardiac defibrillators (operation times of several months to years), while neurostimulators (operation time of a few months) demand power in the range of a few microwatts to milliwatts, and 100–1000 μW are necessary to power drug pumps (lifetime of a few hours).^[^ ^144^, ^145^ ^]^ However, further research is needed to boost the capacities and operation times of transient batteries so that they can power more sophisticated devices similar to conventional batteries, e.g., cochlear implants (20–40 mW, months to years of operation time),^[^ ^145^ ^]^ retinal stimulators (250 mW),^[^ ^146^ ^]^ actuators (≈50–100 mW),^[^ ^147^ ^]^ or portable electronic devices (0.5–12 W),^[^ ^148^ ^]^ which usually operate in the potential range of 1.5–3.0 V.

## 4 Challenges and Future Directions

**Figure 9:**
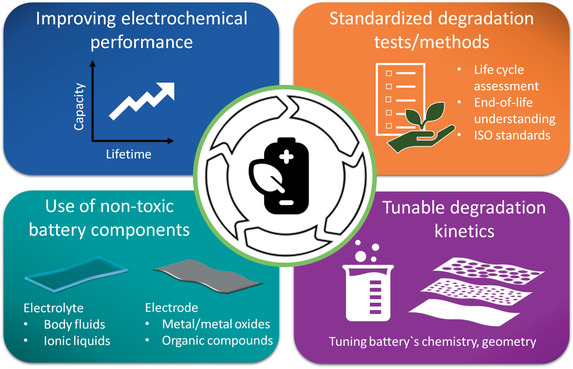
Schematic representation of the main benefits arising from transient batteries and their current challenges toward practical implementation.

While substantial efforts have been devoted to the development of transient electronics in general, transient batteries remain relatively unexplored. It is thus essential that researchers also focus their attention on transient energy storage devices, where technology interacts with nature to leave no permanent footprint. **Figure** **9** schematically depicts the most significant benefits arising from transient batteries and the related future challenges toward practical implementation. Although nowadays transient batteries cannot replace the traditional batteries in certain applications such as those dedicated to large‐scale energy storage systems or electric vehicles, transient batteries can displace the use of durable and long‐lasting batteries in low‐power applications. This substitution of traditional batteries may pave the path to develop eco‐friendly devices that reduce the environmental burdens associated with electronic waste, landfill space, and hazardous components. Even though areal capacities up to 3 mAh cm^−2^, specific energy densities up to 4.70 mWh cm^−2^, and working voltages above 2.0 V have been obtained so far, many of the reported transient batteries suffer from relatively low power density and short lifetime.^[^ ^2^, ^3^, ^12^ ^]^

Given the diverse fields of potential applications, transient batteries must meet many requirements, ranging from negligible toxicity of the degradation products, when intended for biomedicine, to a rapid transiency for security devices. To date, water‐soluble polymers such as PVA, biodegradable polymers such as PLA, PCL, polyanhydride, or biodegradable and renewable polymers including silk fibroin, cellulose, gelatin, chitosan, and sodium alginate have been applied in transient batteries. Plant‐based polysaccharides (cellulose, alginate) and animal‐based polymers (silk, chitosan) are enzymatically degradable, although their batch‐to‐batch variability makes their degradation slightly unpredictable.^[^ ^149^, ^150^, ^151^ ^]^ On the other hand, synthetic biodegradable materials such as polyesters present easy tunability of their degradation profile. However, their biocompatibility should be examined from the raw materials to the final product considering all processing steps.^[^ ^67^ ^]^ Interestingly, besides their biodegradability, many of those polymers are also biosourced, meaning that they are obtained from renewable resources (ISO 16620‐2 standard can be followed to determine the biobased content of solid, liquid, and gaseous samples using carbon‐14 analysis). Polymers that undergo selective depolymerization back to their initial constituent's feedstock may provide novel approaches for the valorization of transient batteries.^[^ ^152^, ^153^ ^]^ This may reduce the environmental footprint related to the intensive use of fossil‐based raw materials while limiting greenhouse gas emissions.

**Figure 10:**
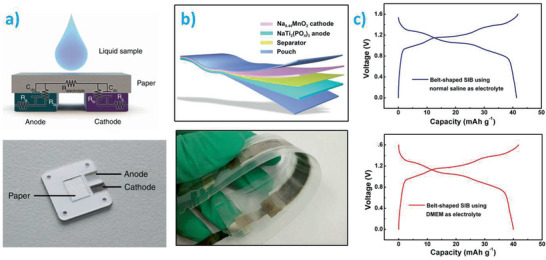
Body‐fluid‐activated batteries: a) scheme showing the generally followed approach in body‐fluid‐activated batteries together with an optical photograph of the battery. Reproduced with permission.^[^ ^155^ ^]^ Copyright 2019, Springer Nature. b) Scheme and optical photograph showing the structure and macroscopic appearance of fabricated Na_0.44_MnO_2_//NaTi_2_(PO_4_)_3_@C battery. c) Electrochemical performance of a Na_0.44_MnO_2_//NaTi_2_(PO_4_)_3_@C battery comprising normal saline or cell culture medium electrolytes; Dulbecco's modified Eagle's medium (DMEM) was used as the cell‐culture medium. b,c) Reproduced with permission.^[^ ^159^ ^]^ Copyright 2017, Elsevier.

To avoid toxicity issues arising from electrolyte leakage of batteries using organic electrolytes,^[^ ^154^ ^]^ body fluids such as gastric juice, urine, saliva, blood, or sweat have been directly applied as electrolytes. As schematically shown in **Figure** **10**a, body‐fluid‐activated batteries are typically constructed from an oxidizing electrode (anode) and a reducing electrode (cathode) physically connected by a hydrophilic, but electronically insulating material such as cellulose.^[^ ^155^ ^]^ Because of its abundant polar —OH groups, cellulose is usually applied as the separator, which upon contact with fluids acts as battery electrolyte. Generally, these batteries cannot be recharged as they stop functioning as soon as the body fluids acting as electrolytes get exhausted. One of the earliest attempts using body‐fluid‐based electrolytes instead of the conventional flammable organic or corrosive electrolytes was reported by Lee in 2005, who built a human‐urine‐activated microbattery composed of a magnesium anode and a copper‐chloride‐doped paper cathode.^[^ ^156^ ^]^ When the urine is dropped onto the paper between the Mg and CuCl layers, the CuCl reacts to form MgCl_2_ and Cu, delivering a total power of 1.5 mW with a maximum operating voltage of 1.4 V, which is enough to drive urine screening on‐board biosensors. The battery could be reactivated again upon urine soaking. More recently, a sweat‐activated primary battery was assembled using a Mg anode, a Ag/AgCl cathode, and a cellulosic separator encased within an elastomeric microfluidic system with multiple outlets to expel excess sweat, allow fresh sweat to enter, and permit the release of hydrogen gas as a product.^[^ ^157^ ^]^ With ionic conductivity values ranging from 1 to 10 mS cm^−1^, the sweat, containing naturally excreted dissolved salts, provides the adequate aqueous electrolytic conditions for closing the circuit. However, the separator of this battery required an impregnation step with NaCl to ensure enough ionic conductivity due to changes of the sweat composition. A maximum operating voltage of 1.8 V was obtained, resulting in a specific capacity of 67 Ah kg^−1^ and capable of operating for 5 h. As a representative example of a transient battery powering an in vivo device, a gastric‐fluid‐activated biocompatible battery comprising a Zn anode and a Cu cathode was reported.^[^ ^158^ ^]^ During operation, Zn undergoes galvanic oxidation and the inert Cu cathode returns the electrons to the solution. The cell delivered an average power of 0.23 μW mm^−2^ for 6.1 days to support a temperature sensing and wireless communication system located within the gastrointestinal tract of pigs.

Despite these relevant works, scarce efforts have been done to design a rechargeable/secondary battery working with body‐friendly fluids. In this context, Guo et al. reported a secondary sodium‐ion battery functioning with normal saline (0.9 wt% NaCl) or a cell‐culture medium mimicking the fluid present around cells in the human body (having amino acids, sugars and vitamins).^[^ ^159^ ^]^ As shown in Figure 10b, a Na_0.44_MnO_2_ cathode, a NaTi_2_(PO_4_)_3_@C anode, and a microporous polyacrylonitrile separator were used. The battery showed a specific capacity of 41 and 40 mAh g^−1^ at a current density of 0.2 A g^−1^ for normal saline or cell‐culture medium, respectively (see Figure 10c). Moreover, O_2_ in the electrolyte was consumed during discharge, showing promising application in implantable electronic devices for cancer starvation therapy.

Even though the use of internal biofluids as battery electrolytes is an interesting strategy, it should be considered that the constantly changing nature of biofluids can have undesirable effects on the resulting electrochemical performance. The pH, chemical composition, and viscosity of gastric fluids can vary throughout the day, while the Na^+^ concentration in sweat depends on the sweat rate,^[^ ^160^ ^]^ thus influencing the ionic conductivity and the battery performance. Therefore, it is not at all trivial to design a transient battery that can ensure a constant electrochemical environment inside the cell, especially if the battery is located within a constantly changing human body.^[^ ^33^ ^]^ ILs, nonvolatile room‐temperature molten salts, represent other sustainable electrolyte choices as they are highly conducting and present good biocompatibility.^[^ ^161^, ^162^ ^]^

Additionally, bioresorbable (magnesium), biocompatible (iron, tungsten, molybdenum), or dissoluble (vanadium pentoxide, as it dissolves in the alkali solution formed when Li metal reacts with H_2_O to form LiOH)^[^ ^2^ ^]^ metals/metal oxides, or biologically derived compounds (melanin, quinones) are found to be suitable as electrodes because of their capability to reversibly bind/host different ions. Alloy composition and microstructure design may help to obtain electrodes with limited parasitic corrosion.^[^ ^99^ ^]^ Although pure metals and metal oxides are the most common materials to develop batteries, primary batteries with continuous operation for 100 min using solely organic materials are possible.^[^ ^141^ ^]^

In comparison to the many efforts carried out to develop transient batteries for the biomedical field, scarce attempts have been made to develop high‐power biodegradable batteries for environmental applications. In this regard, Esquivel et al. developed a biodegradable flow battery based on cellulose, carbon paper, beeswax, and organic redox species.^[^ ^141^ ^]^ With an output voltage and power of 3.0 V and 2.8 mW, respectively, the battery could be disposed in an organic waste container. After 60 days, 54 ± 4% of the battery weight was biotically degraded under standardized anaerobic conditions (the worst‐case scenario for biodegradation).

Intense endeavors are now focusing on the reuse, remanufacturing, and recycling of batteries to expand their useful life as a strategy to reduce the potential harmful effects of batteries on human health and the environment.^[^ ^163^, ^164^, ^165^ ^]^ As conventional batteries usually require a high‐tech treatment for effective recycling, transient batteries can facilitate material recovery, enabling cheaper and more efficient recycling processes. In a complementary manner, transient batteries can provide a suitable solution to the accumulation of dangerous electronic waste that is dumped inadequately, significantly improving environment and people's health in the future.

Bearing this in mind, it is evident that transient batteries have a huge potential to facilitate the ecological transition by introducing a new model based on circularity. In this context, in 2015, the United Nations General Assembly established 17 Sustainable Development Goals (SDGs) which aim to promote prosperity while protecting the planet.^[^ ^166^ ^]^ Due to their inherent degradable character, transient batteries have a particular potential to progress on several SDGs. Transient batteries may contribute to the “SDG 7: Affordable and clean energy” by making widely available the access to more sustainable energy storage systems. Lowering our dependence on the use of nonrenewable resources that have a destructive impact on the biosphere, transient batteries have the potential to achieve “SDG 12: Responsible consumption and production.” Finally, the use of transient batteries may protect the environment against the disposal of durable or toxic materials, protecting “Life below water” and “Life on land,” SDGs 14 and 15, respectively.

In this review, we highlight that the transiency depends on both the nature of the material itself (chemical and crystalline structure, morphology) and the environmental conditions of the medium (temperature, pH, etc.). Therefore, if one can foresee the degradation conditions of the battery, it should be possible to precisely engineer the battery design to obtain a specific degradation time frame. Controlling the geometry of battery components (thickness, surface area, and morphology) may allow a fine‐tuning of the kinetics of the battery transiency. For example, longer‐lasting transient batteries are achieved by increasing encapsulation thickness or by decreasing the exposed surface area (no porosity). The chemistry of the device also plays a major role in the transiency, as chemical cross‐linking of the encapsulation/separator or surface modification (to render the material hydrophobic) may lower the susceptibility of the material to undergo chain‐cleavage reactions. Moreover, the crystal structure of the materials itself can be tuned to obtain tailored degradation behavior, as the degradation rate in the aqueous environment of biopolymers such as silk is dependent by several orders of magnitude on its crystalline *β*‐sheet structure.^[^ ^88^ ^]^ Another aspect worthy of noticing is that the chemistry of the battery components (anode, cathode, separator/electrolyte, current collector, and packaging) could be tailored in such a way that the battery operates normally until the introduction of specific triggers such as water, pH change, exposure to light, or temperature initiate the degradation process. In any case, one should take into account that focusing exclusively on the transiency may lead to inferior battery performance. Therefore, researchers must find an adequate balance in the trade‐off between device´s electrochemical performance and transient behavior.

Despite significant progress, a lot of work remains to be carried out to better understand the life cycle (especially the EOL) of materials that are being developed for transient batteries, in particular those for the anodes and cathodes. This applies not only to pure elements (i.e., metallic lithium, cobalt, iron, etc.) but also to their chemical compounds (LiCoO_2_, lithium iron phosphate, etc.) as these compounds will be found together in an actual device. It should be taken into account that not all biodegradable materials undergo degradation processes in all environments, i.e., some may biodegrade in waste processing facilities, while others may do better in soils.^[^ ^167^, ^168^ ^]^ Moreover, a biodegradable material does not per se mean that it is biosourced, compostable, or renewable. To date, the transiency of batteries has mainly been proven under simple in vitro tests based on hydrolytic degradation, either in deionized water or in simulated biofluids like PBS. However, to get a better picture, it is essential to evaluate their degradation not only in PBS, an in vitro surrogate of physiological fluid at 37 °C, but also in different body fluids such as gastric acid, saliva, blood, or urine. It is important to note that in vivo degradation behavior can be very different from in vitro behavior. For some polymers such as poly(trimethylene carbonate) or poly(*ε*‐caprolactone), in vivo degradation occurs much faster than in vitro degradation due to the action of enzymes.^[^ ^169^, ^170^ ^]^ The presence of complex compounds such as proteins, but even simple ions can lead to unforeseen results comparing with in vitro studies. For example, calcium ions can accelerate the dissolution rates of silicon nanomembranes, while proteins show just the opposite effect.^[^ ^171^ ^]^ Therefore, in vivo degradation analyses offer the most compelling approach to assess the degradation behavior and potential risks of transient batteries. It is also worth mentioning that although the biocompatibility of different inorganic materials used in transient batteries has been evaluated through cell toxicity tests and animal models, their environmental impact has not been investigated yet. To examine the potential environmental impact, future research works on transient batteries should focus their efforts on the analysis of the biodegradation process according to harmonized standards such as ISO 14855‐1:2012, ASTM D5338, ASTM D6868, ASTM D7044, ASTM D8029, or CEN EN 13432:2000. However, as transient batteries might be used in a wide variety of environments, even the benefit of standardized tests can be somewhat limited.

Besides, the determination of the life cycle environmental impacts of batteries through methodologies such as life cycle assessment may enable a better quantification of their contribution to ozone layer depletion, abiotic depletion, global warming, or eutrophication.^[^ ^172^ ^]^ This would help to foresee efficient management of the EOL of transient batteries, establishing policies that promote their efficient biodegradability.

Additionally, in vivo degradation studies of transient batteries may further deepen the understanding on how different battery components interact/behave in real biological solutions or what maximum dosage of materials is allowed to prevent threat to the human body. Those studies have revealed, for example, that hydrogen gas formation during battery degradation can lead to undesirable tissue necrosis and blood clotting (in vitro studies may just show that the battery is degradable). The degradation of a battery in PBS was compared with in vivo degradation in the subcutaneous area of Sprague‐Dawley rats during 4 weeks.^[^ ^12^ ^]^ Batteries for in vivo applications require nontoxic degradation products that are assimilated by the body through bioabsorption, phagocytosis, or metabolization processes. Particularly, fundamental operando studies under biological conditions analyzing both the battery performance and their interaction with biotic systems (tissues and organs) are required. A demonstration of fungal biodegradation of transient batteries so that they can be applied as forestry fertilizers should also be developed.^[^ ^173^ ^]^ These results may shed light on the design and fabrication of transient batteries for biomedical and environmental applications.

Ideally, transient batteries are envisaged as an additional opportunity to provide novel functionalities to the degrading medium. This can be achieved by incorporating additives into transient devices that can be then released in a controlled fashion during the EOL of the battery, enriching the environment. For example, silk fibroin releases amino acids during its degradation,^[^ ^89^ ^]^ which can be used for agricultural or cosmetic purposes. Particularly interesting chemistries are zinc‐ion batteries, which contain a high capacity zinc metal as anode and safe mildly acidic or neutral water‐based electrolytes (pH 3–7).^[^ ^174^ ^]^ Zinc is cheap, highly abundant, and represents an essential element for cells as it acts as an intracellular secondary messenger in several cellular processes.^[^ ^175^ ^]^ As a matter of fact, a zinc anode has been combined with a Pd cathode to fabricate a gastric battery for a wireless endoscopy application, highlighting the potential of zinc‐ion batteries for implantable applications.^[^ ^176^ ^]^ Mg batteries represent another interesting option, which, using body fluids as electrolytes, can yield a theoretical voltage of 3.09 V and an energy density of 2840 Wh kg^−1^.^[^ ^177^ ^]^ In any case, it should be noted that pure magnesium suffers from quick corrosion in aqueous solutions, so more resistant Mg alloys such as AZ31 (96 wt% Mg, 3 wt% Al, and 1 wt% Zn) are desired.^[^ ^32^ ^]^

Although the debate about whether recyclable or biodegradable is the best for the environment is ongoing, without any doubt, biodegradable transient batteries represent a step forward in developing more sustainable alternatives to standard batteries. Transient batteries provide us an opportunity to change the current paradigm which considers batteries as harmful waste, to use them as nurture for the environment. Transient batteries hold a promising future to be a frontrunner in the uptake of circular economy concepts, opening new and highly innovative application fields.

## Conflict of Interest

The authors declare no conflict of interest.
